# Quantitative systems pharmacology of interferon alpha administration: A multi-scale approach

**DOI:** 10.1371/journal.pone.0209587

**Published:** 2019-02-13

**Authors:** Priyata Kalra, Julian Brandl, Thomas Gaub, Christoph Niederalt, Jörg Lippert, Sven Sahle, Lars Küpfer, Ursula Kummer

**Affiliations:** 1 Department of Modelling of Biological Processes, COS/BioQuant, Heidelberg University, Im Neuenheimer Feld 267, Heidelberg, Germany; 2 Now at Department of Systems Biology, Technical University of Denmark, Kgs. Lyngby, Denmark; 3 Clinical Sciences, Bayer Pharma, Kaiser-Wilhelm-Allee 1, Leverkusen, Germany; Harvard Medical School, UNITED STATES

## Abstract

The therapeutic effect of a drug is governed by its pharmacokinetics which determine the downstream pharmacodynamic response within the cellular network. A complete understanding of the drug-effect relationship therefore requires multi-scale models which integrate the properties of the different physiological scales. Computational modelling of these individual scales has been successfully established in the past. However, coupling of the scales remains challenging, although it will provide a unique possibility of mechanistic and holistic analyses of therapeutic outcomes for varied treatment scenarios. We present a methodology to combine whole-body physiologically-based pharmacokinetic (PBPK) models with mechanistic intracellular models of signal transduction in the liver for therapeutic proteins. To this end, we developed a whole-body distribution model of IFN-*α* in human and a detailed intracellular model of the JAK/STAT signalling cascade in hepatocytes and coupled them at the liver of the whole-body human model. This integrated model infers the time-resolved concentration of IFN-*α* arriving at the liver after intravenous injection while simultaneously estimates the effect of this dose on the intracellular signalling behaviour in the liver. In our multi-scale physiologically-based pharmacokinetic/pharmacodynamic (PBPK/PD) model, receptor saturation is seen at low doses, thus giving mechanistic insights into the pharmacodynamic (PD) response. This model suggests a fourfold lower intracellular response after administration of a typical IFN-*α* dose to an individual as compared to the experimentally observed responses in *in vitro* setups. In conclusion, this work highlights clear differences between the observed *in vitro* and *in vivo* drug effects and provides important suggestions for future model-based study design.

## Introduction

Pleiotropic interferon alpha (IFN-*α*) belongs to the type I IFNs family. IFN-*α* is an extensively used cytokine in clinical medicine, effective in hepatitis C (HCV) and hepatitis B (HBV) treatment over the past 20 years [1–9]. Despite its routine application in clinical practice [10], there is incomplete understanding regarding its modes of action and the causality of induced pharmacodynamic effects. Therefore, hepatocytes have become important *in vitro* study models for IFN-*α* action [11]. One hindrance to discern the molecular response in hepatocytes to IFN-*α* treatment is that the experimental investigation requires liver biopsies of patients undergoing IFN-*α* therapy. This is ethically difficult, if not infeasible and would impose a significant burden for the patient [12].

**Fig 1:**
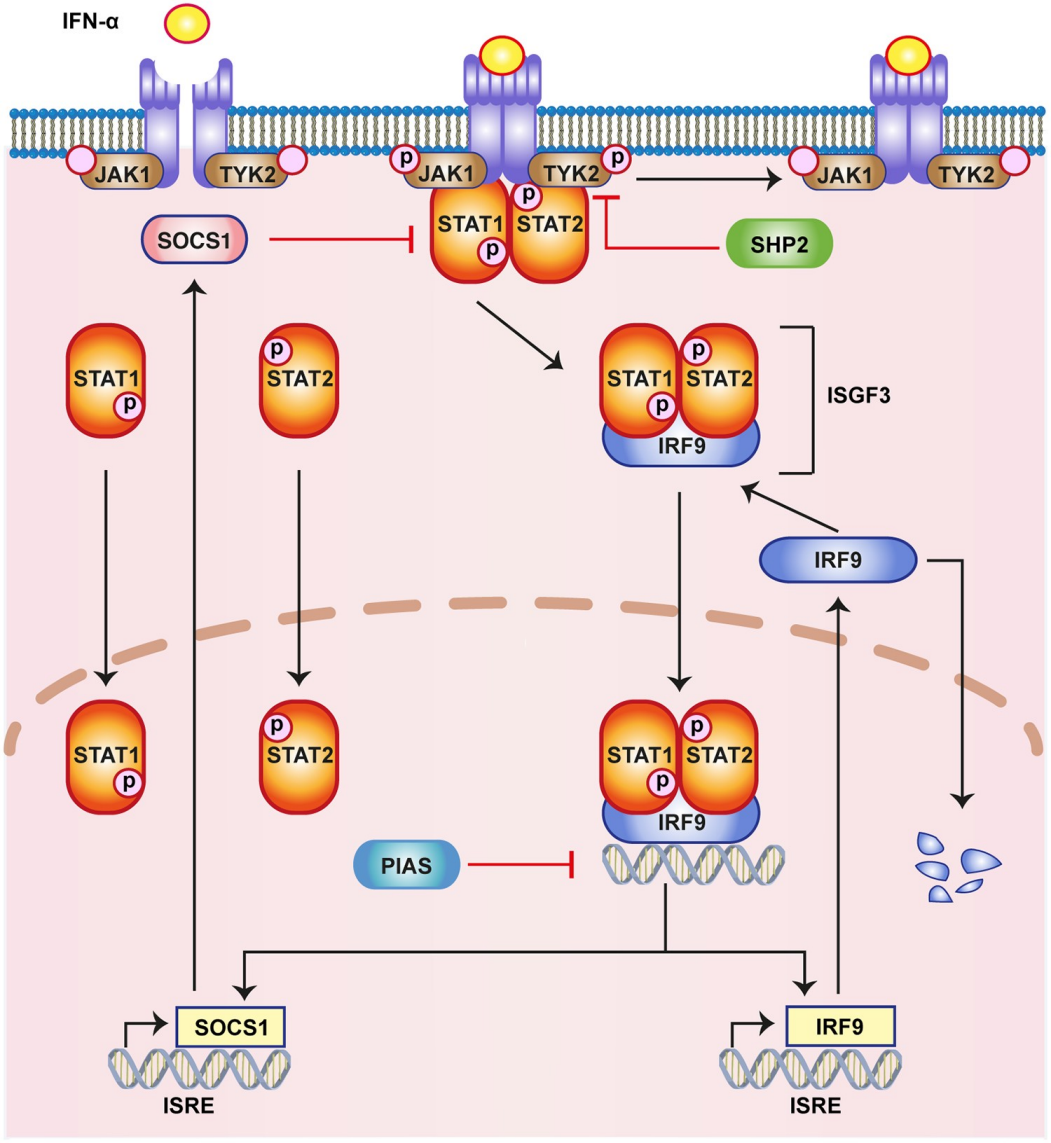
Illustration of the canonical JAK/STAT pathway. The key steps of the pathway in response to IFN-*α* are highlighted here. IFN binds to the receptor and activates JAK1 and TYK2, which subsequently activate the STATs in the cytoplasm, all by phosphorylation. The phosphorylated STATs and IRF9 form the transcription complex ISGF3, which translocates to the nucleus, where it activates hundreds of ISGs, including IRF9, Mx2 and SOCS1.

IFN-*α* canonically acts via the JAK/STAT pathway (Fig 1). IFN binds to the interferon receptor subunits IFNAR1 and IFNAR2 to form a heterodimeric ligand receptor complex. This heterodimeric ligand receptor complex activates intracellular signalling via the receptor associated kinases Tyk2 and JAK1, which mutually phosphorylate each other. STAT1 and STAT2 molecules associate with the receptor complex and form a phosphorylated hetero-dimer. The phosphorylated heterodimer of pSTAT1/2 is released from the receptors to form the hetero-trimeric ISGF3 transcription factor by binding IRF-family member IRF9 (p48/ISGF3). ISGF3 translocates into the nucleus and activates the interferon-stimulated response elements (ISRE). The activated ISREs lead to the expression of various antiviral proteins like Mx1, Mx2 and RNA-dependent protein kinase (PKR) [13]. The pathway exerts several negative feedbacks that result in a strong regulation of the interferon-stimulated genes (ISGs). SOCS (suppressors of cytokine signalling), PIAS (protein inhibitors of activated stats) and PTPs (protein tyrosine phosphatases) are among the most important negative regulators of the pathway. Genes for SOCS and IRF9 can contain both GAS and ISRE as transcription factor binding sites and are therefore activated by IFN dependent signalling.

On the pharmaceutical side, IFN-*α* pharmacokinetics are fairly well described. A significant inter-individual variability is well known from clinical practice [14, 15]. In the future, personalised treatment scenarios might arise from the deeper understanding of the detailed mechanisms of IFN-*α* action and inter-individual patient variability, improving success rates of IFN-based therapies [16–18]. In order to gain an integrative and profound understanding of IFN-*α* drug action, we use a quantitative systems pharmacology (QSP) approach which simultaneously considers multiple levels of biological organisation [19]. For this purpose, we integrated a detailed cellular model of IFN-*α* signalling through the JAK/STAT pathway [20] into a physiologically-based pharmacokinetic (PBPK) model at the whole-body scale [21, 22] for healthy humans. Thus, it was possible to simultaneously describe IFN-*α* pharmacokinetics as well as the resulting drug-induced response, i.e. the pharmacodynamics (PD), within one integrated multi-scale model representation.

**Fig 2:**
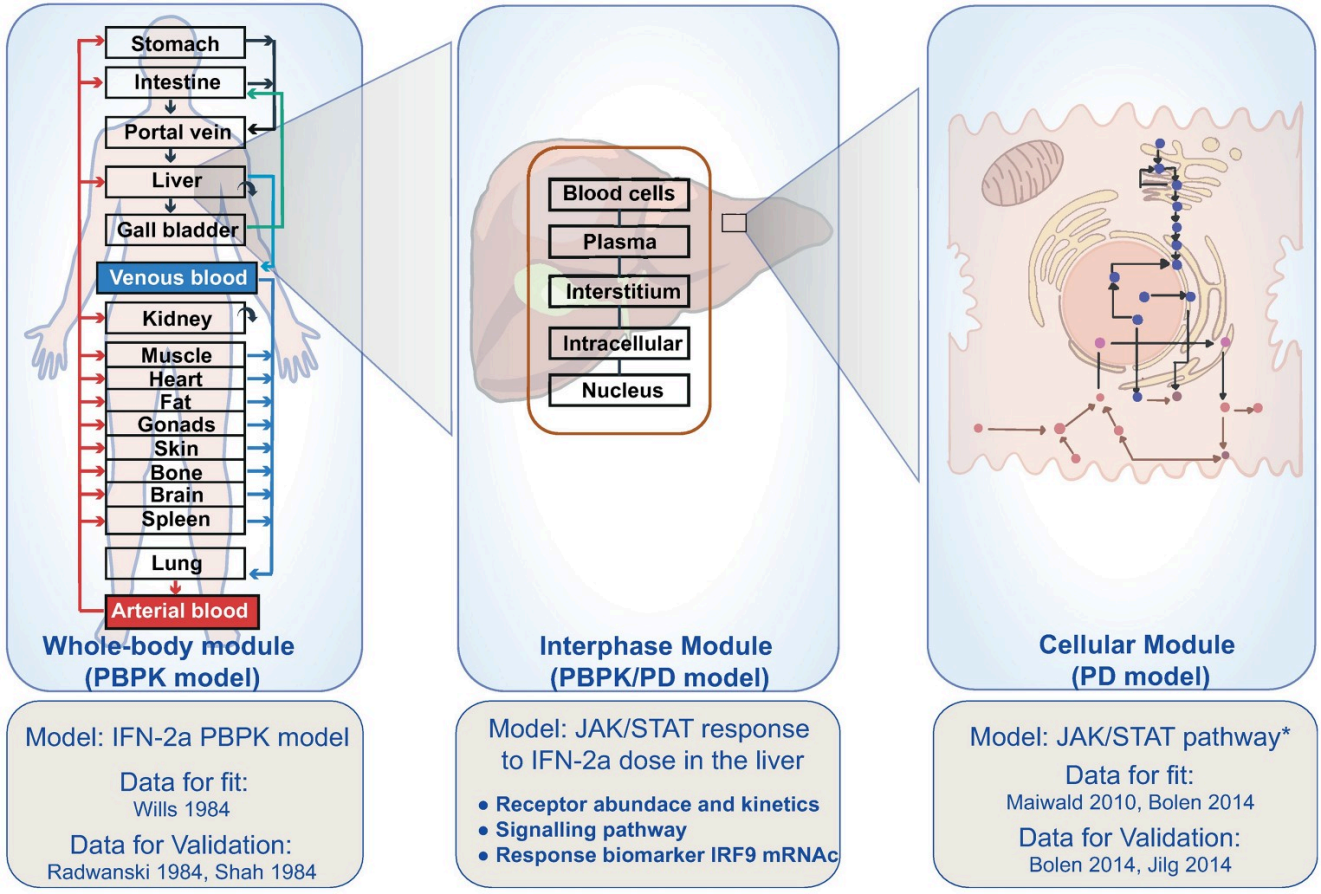
Integrated multi-scale PBPK/PD model. From left to right: Whole-body model (PBPK): The drug distribution is modelled using the PBPK approach at the level of the human whole-body. Integrated model (PBPK/PD): The integrated model encompasses the dose arriving from the whole-body PBPK model to the liver and the cellular model coupled to the PBPK model inside the liver. The target mediated drug disposition is modelled in the liver compartment of the PBPK model. It allows for organ level analysis after the whole-body drug distribution. Here, the biophase distribution is taken into account for large molecules. Cellular model (PD): The cellular model established by using data from hepatocytes consists of the mechanism based PD model comprising the receptor behaviour and signalling network. This model is an altered version of the model from [20] which is then coupled at the liver with the whole-body PBPK model.

Computational models of intracellular signalling pathways have been previously developed to describe IFN-*α* induced responses in the cellular signalling network [20, 23–28]. To calibrate these non-linear ODE-models, quantitative data which are primarily generated under controlled *in vitro* conditions such as cell cultures (reviewed in [29]) or tissue cultures [30], are needed. However, many *in vitro* assays only allow stationary concentration profiles in contrast to the highly dynamic PK concentration profiles occurring *in vivo*. On the contrary, the integration of cellular models within whole-body PBPK models provides the unique opportunity to simulate cellular responses to IFN-*α* stimuli within an *in vivo* context. We present a multi-scale PBPK/PD model that simultaneously quantifies the amount of IFN-*α* reaching the liver in a time-resolved manner, as well as the induced response in the intracellular signalling network. Ultimately, administration of IFN-*α* leads to the expression of IRF9 mRNAc (Fig 2). To our knowledge, thus far, this is the first study on therapeutic proteins that integrates the models of different scales in such detail. We expect such multi-scale PBPK/PD models to become of increasing importance in pharmaceutical development in the future.

## Materials and methods

### Software

#### Copasi

The cellular JAK/STAT signalling model of IFN-*α* was developed in Copasi [31], version 4.22, an open source software freely available at www.copasi.org.

#### PK-Sim

The PBPK model of IFN-*α* was built using the PBPK modelling software PK-Sim^®^ (Open Systems Pharmacology suite Version 7.0 available at www.github.com).

#### MoBi

The PBPK protein model was established in PK-Sim (Open Systems Pharmacology suite Version 7.0). The integration of the PBPK model with the cellular JAK/STAT signalling model was implemented in MoBi^®^ (Open Systems Pharmacology suite Version 7.2 available at www.github.com).

### Methods

Simulation of the models were performed using the LSODA algorithm as implemented in Copasi, as well as cvode 42 in PK Sim/MoBi. Parameter fitting was performed with the Particle Swarm and Hooke/Jeeves algorithms as implemented in Copasi for fitting intracellular dose-response behaviour and Monte Carlo/Levenberg-Marquardt implemented in the Open Systems Pharmacology suite (Matlab toolbox).

#### PBPK modelling

PBPK modelling allows a detailed mechanistic representation of physiological processes underlying drug ADME (ADME: absorption, distribution, metabolism and excretion) at the whole-body level. PBPK models are based on physiological information of the organism such as organ volumes or blood flows on the one hand and physicochemical properties of the drug, i.e. its molecular weight or lipophilicity on the other. In total, a PBPK model may comprise several hundreds of ordinary differential equations and a corresponding number of model parameters. However, physiological information on the organism is usually compiled in curated databases in most PBPK software parameters such that these parameters do not have to be provided by the user. In a PBPK model each organ is characterised by a blood perfusion rate or organ surface area. The various organs are linked together by arterial and venous blood compartments to allow for vascular circulation at the whole-body scale. In addition physicochemical parameters are required to parametrise the distribution model to simulate steady state tissue concentrations as well as the corresponding passive permeation rates. Notably, the distribution of both small molecules (MW 400 g/mol) and of proteins (MW 100,000 g/mol) can be considered with PBPK modelling. The only difference in this case is an adjusted distribution model.

## Results

### Model set-up

The multi-scale model was obtained by establishing and merging the models as in the following steps:

1. Establishment of a PBPK model of interferon administration at the whole-body level.
2. Establishment of a revised intracellular PD model of the JAK/STAT pathway at cellular scale.
3. Integration of both models in a multiscale PBPK/PD model of IFN-*α* drug action.

In the following we walk through the individual steps in detail.

### Establishment of a PBPK model of interferon administration at the whole-body level

For the PBPK of IFN-*α* weight, height and BMI of an average European individual were selected in the biometrics section of the individual. Physico-chemical information of IFN-*α* was obtained from the literature (S4 and S5 Tables).

The ligand-receptor interaction kinetics were represented according to the established kinetic mechanisms described in previous studies [32–34] (S2 Text). The relative protein abundance of IFNAR2 and IFNAR1 for their tissue distribution expression profiles of normal, healthy individuals was taken from an *in vivo* expression database [35]. In the PBPK model both receptors were assumed to occur in the interstitial space of the liver and the individual receptor concentrations were calculated according to the hepatocellularity of the human liver (S2 Text). For estimating the kidney clearance, elimination values by glomerular filtration rate (GFR) were inserted from the literature (S4 Table).

**Fig 3:**
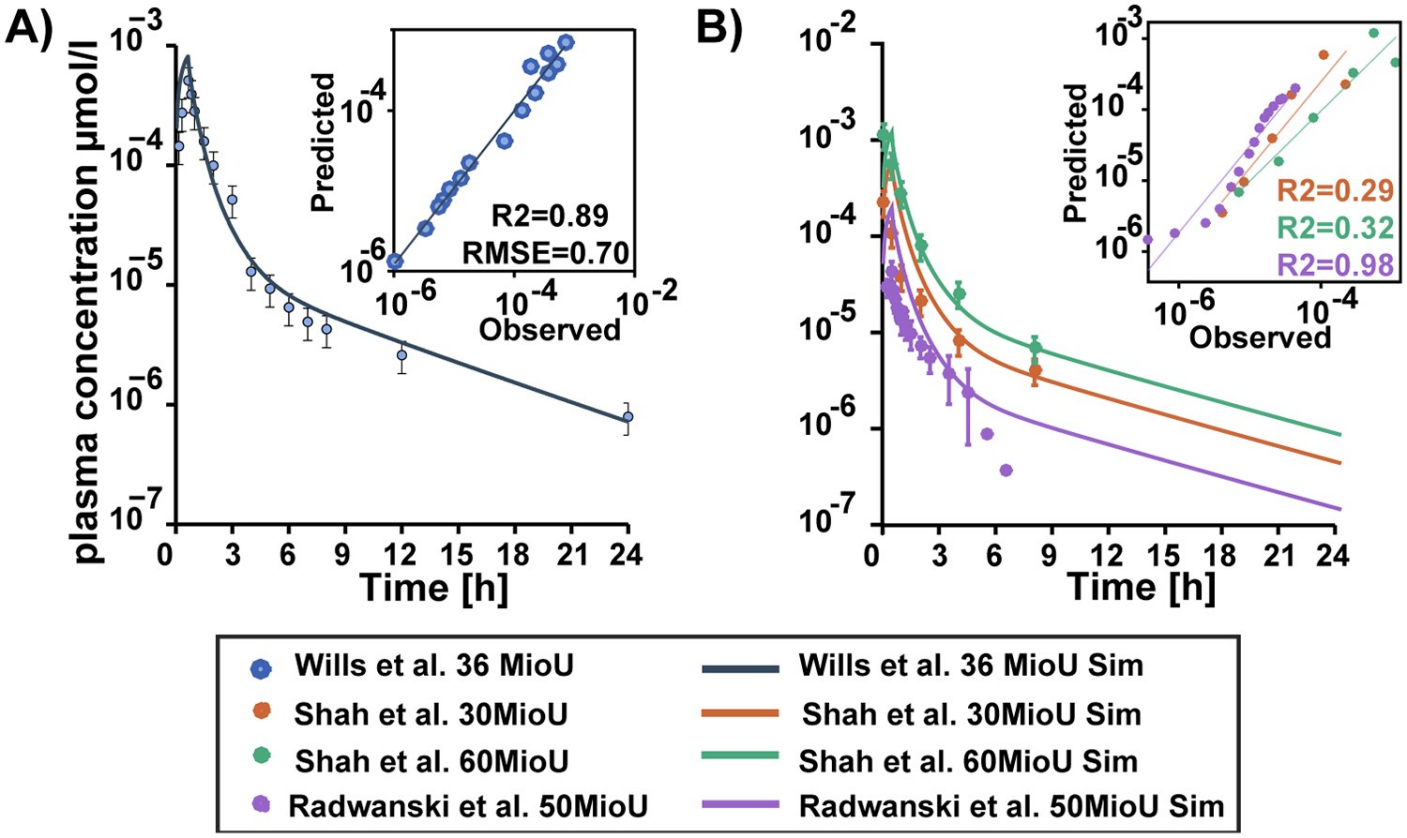
Physiologically based pharmacokinetic (PBPK) model fit and validation. Physiologically based pharmacokinetic (PBPK) model simulations (lines) and experimental blood plasma profile (circles) of IFN-*α* in humans. A) Experimental results of Wills et al. [36] and the corresponding fit. B) Model validation using blood plasma profile experiments from literature [37, 38].

Intravenous administration of recombinant leukocyte IFN-*α* of 0.22 mg on human subjects as reported by Wills et al. (1984) [36] was used to fit the model. These data served as the basis to estimate parameters like binding affinity and internalisation to replicate the ADME profile represented therein (details in the Supplement S2 Text). Validation was done by using other datasets from literature, namely: Intravenous IFN-*α* doses 0.136 mg and 0.27 mg by Shah et al.(1984) [37] and 0.045 mg by Radwanski et al. (1987) [38] (see Fig 3A and 3B). The model describes these data with reasonable accuracy (see S6 Table).

PBPK models can quantify the tissue exposure to the drug. In the case of IFN-*α* many tissue distribution studies have been performed on animal models [39–42]. In these mouse studies, highest concentrations have been found in the spleen, liver, kidney and lungs. In this context, the concentration denotes the whole tissue concentration of IFN-*α*.

**Fig 4:**
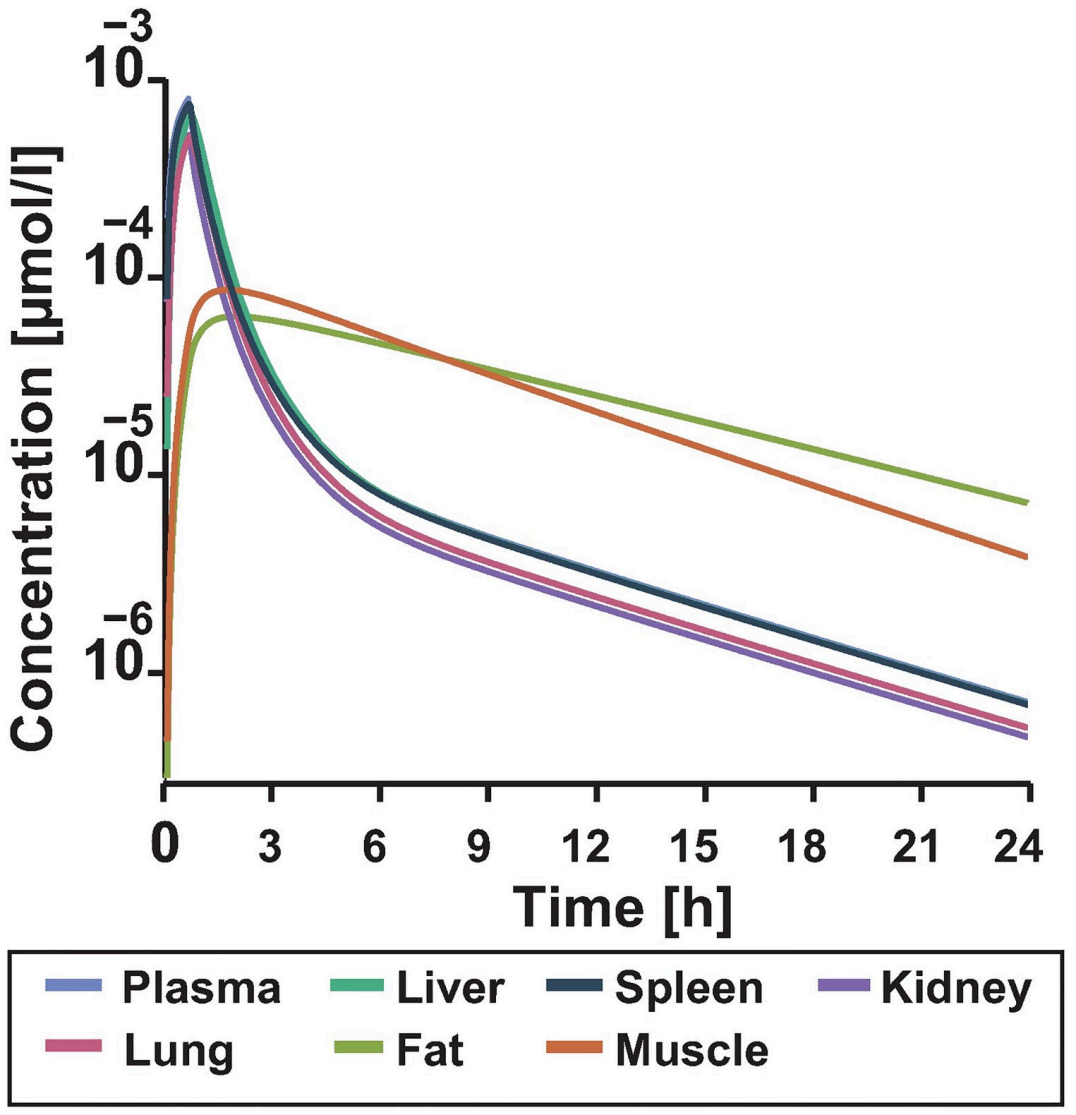
Simulated tissue distribution of IFN-*α* for the dose reported in Wills et al. (1984) [36] for 24 hours. The lines show time-course distribution of IFN-*α* in venous blood plasma vs. the concentration found in the tissue of the different organs.

Tissue distribution studies are rarely possible in humans due to ethical reasons. However, the established PBPK model can give insights into the IFN-*α* tissue concentrations. Our simulation results suggest that the liver, spleen, lung and kidney have similar profiles as the plasma concentration (Fig 4). In particular, the concentration profile in the liver follows the dynamics of the plasma PK profile which has even been shown for higher molecular weight antibodies [22, 43]. On this basis, the PBPK model accurately describes the IFN-*α* concentration in the tissue which is of relevance for the model coupling.

### Establishment of a revised intracellular PD model of the JAK/STAT pathway at cellular scale

The original intracellular model lacked details of the processes at receptor level which are a prerequisite for the model integration into the whole-body model. Therefore, we established an altered version of an IFN-*α* signalling model published by Maiwald et al. [20]. The original model can be retrieved from the Online Cellular Systems Modelling Database [44]. As the receptor level constitutes the interface between PBPK and intracellular model, we extended the original model by a detailed description of these receptor associated processes, using the parametrisation from the PBPK model (S1 Text, S2 and S3 Tables). This resulted in a model of 21 species and 20 reactions (S1 Table).

**Fig 5:**
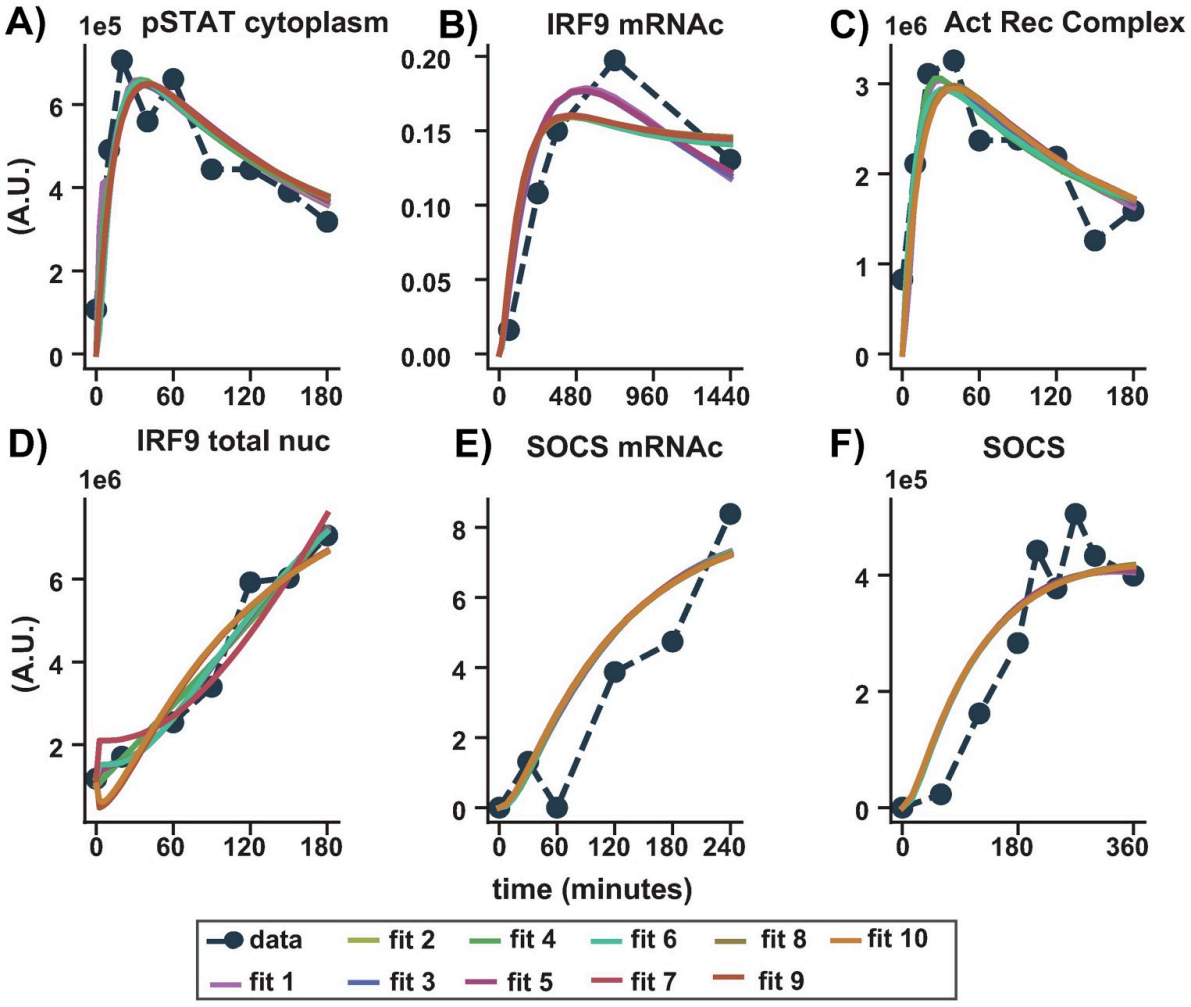
Fitting results of top 10 fits. Comparison between experimental time series measurements of IFN-*α* induced intracellular responses and computational simulations after fitting. The time after IFN-*α* application is given on the x-axis of each subfigure. Experimentally determined levels of the measured proteins are presented as filled dots and dashed lines (A) pSTAT, C) pJAK (Act Rec Complex), D) IRF9 total in the nucleus, E) SOCS mRNAc and F) SOCS protein measured by quantitative-immunoblotting in Huh 7.5 cells after stimulation with 500 U IFN-*α* as in Maiwald et al. [20]. B) dynamic expression of IRF9 mRNA fold change in Huh 7 cells for 10 U IFN-*α* as shown in Bolen et al. [45]. Computational fit shown as solid lines. Fits to full 20 dataset are shown in the supplementary S2 Fig.

In this altered model, the dose response behaviour towards realistic IFN-*α* doses was also established. The original model was fitted to data from close to saturation doses, therefore, we used additional experimental data corresponding to responses to lower doses from the literature. In summary, this model was fitted to the data used in the original publication (Maiwald et al.(2010) [20]) which is data describing the time course of JAK, STAT, IRF9 and SOCS concentration for two hours in a population of Huh 7.5 cells in response to 2 doses: 500 and 1000 Units (U) of IFN-*α* (close to saturation doses). In addition and simultaneously, the following data was fitted: Data from Bolen et al.(2014)m [45] describing the dose response of IRF9 mRNAc for IFN-*α* doses of 10 and 100 U in a population of Huh 7.5 cells. The remaining data from Bolen et al.(2014) [45] for 500 and 2500 U doses and data from Jilg et al.(2014) [46] with dose response behaviour of IRF9 mRNAc for IFN-*α* doses of 15 and 30 U in a population of Huh 7.5 cells were set aside for validation purposes. In addition to the low dose responses, these data sets also capture a longer time course compared to the Maiwald et al. [20] data set. The fitting was achieved with the software COPASI [31] which allows the export of the models in standard SBML format [47]. The fitting procedure was repeated with different initial parameters and the ten best fits were selected for further evaluation of the model. The analysis of these fits, i.e. their parameter distributions showed that almost all parameters remained unidentifiable. Therefore, all fits were studied as a model ensemble to see if results were robust towards parameter variations as in Fig 5 and S1 Fig [48].

**Fig 6:**
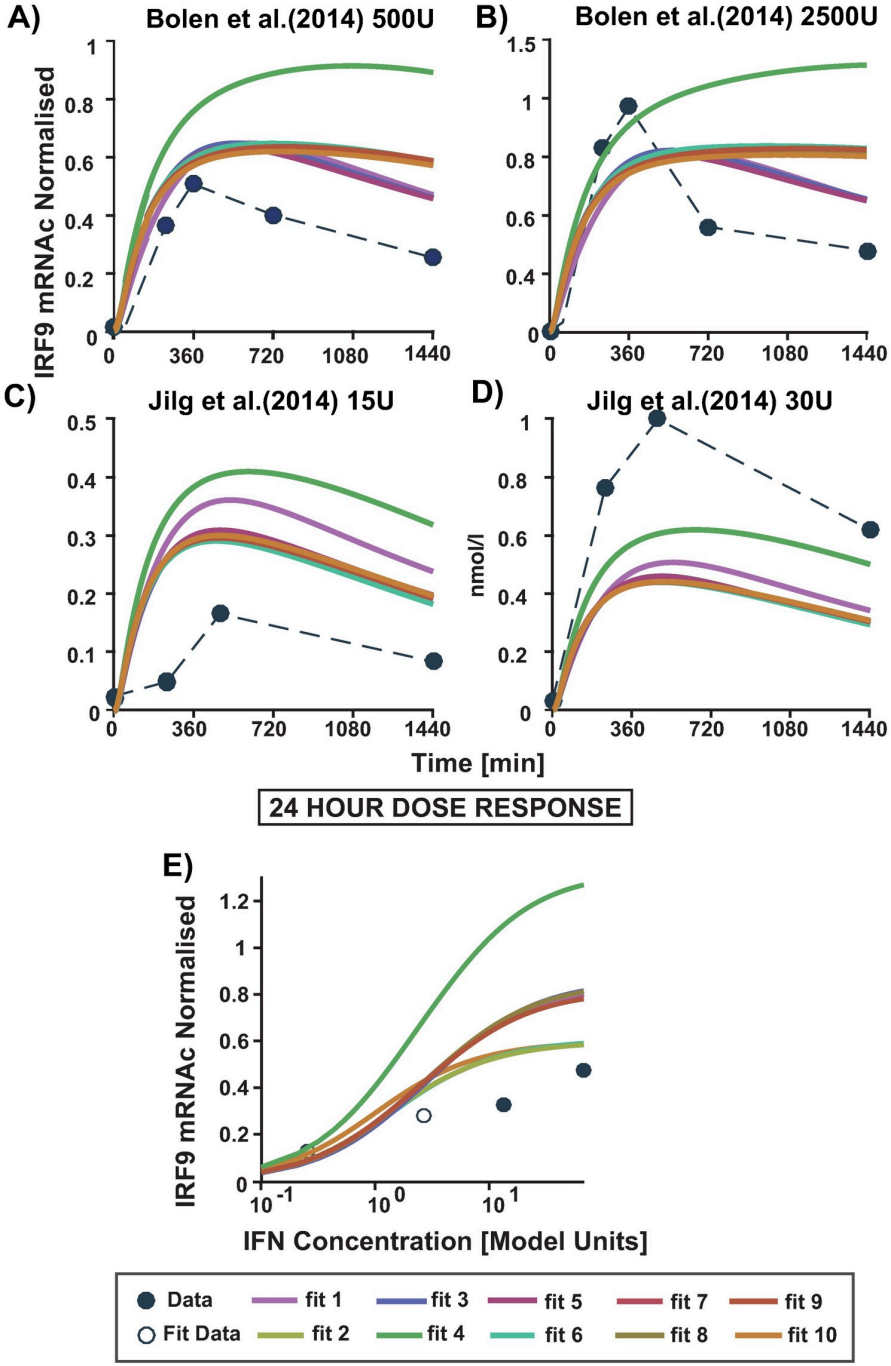
The validation of the dose response for the top ten fits. In A-D) the time course of the 4 experimental doses (circles) is plotted together with the calculated simulation of the top ten models (solid lines). The different doses taken in account are, in A) 500 U and B) 2500U of IFN-*α* as published by Bolen et al. [45]. C) 15U and D) 30U of IFN-*α* as in Jilg et al. [46]. In the panel E) and F) the 24 hour dose response (DR) curve is shown for all the data obtained from the literature where E) has DR simulation for 24th hours representing data from Bolen et al. [45] for 10U, 100U, 500U and 2500U.

As seen in Fig 5 all 10 models are able to fit the experimental data almost equally well. A validation of the response in the model ensemble is done with the doses of 500U and 2500U IFN-*α* from Bolen et al [45] and 15U and 30U IFN-*α* from Jilg et al. [46] as stated previously. As the experiments originate from different publications and are in arbitrary units, they are not directly comparable with each other, but it is possible to compare a normalised relative behaviour of IRF9 mRNAc over time for different doses. This was achieved by normalising across the different doses used in each experiment to account for the fold changes. Hence, having the doses 10U, 100U, 500U and 2500U been normalised across each other, while 15U and 30U having been normalised across the two. It can be seen in Fig 6 that the dynamic behaviour of the model ensemble for 500U and 2500U quantifies the time course data dynamics quite well, although for 2500U the peak concentration is underestimated ((A) and (B)). In Fig 6C) and 6D), the model ensemble overestimates the peak of the time course, but estimates the dynamic behaviour for 15U quite well. However, it also underestimates the response for 30U. It should be noted that in the latter data set a different subtype of interferon was used and the general experimental set up differed. The model shows saturation behaviour already for 100U (hollow circles) which is close to the experimentally estimated saturation dose (between 50U to 100U) (Fig 6E)) as can be seen in the literature (as listed in S3 Fig).

### Integration of both models in a multiscale PBPK/PD model of IFN-*α* drug action

**Fig 7:**
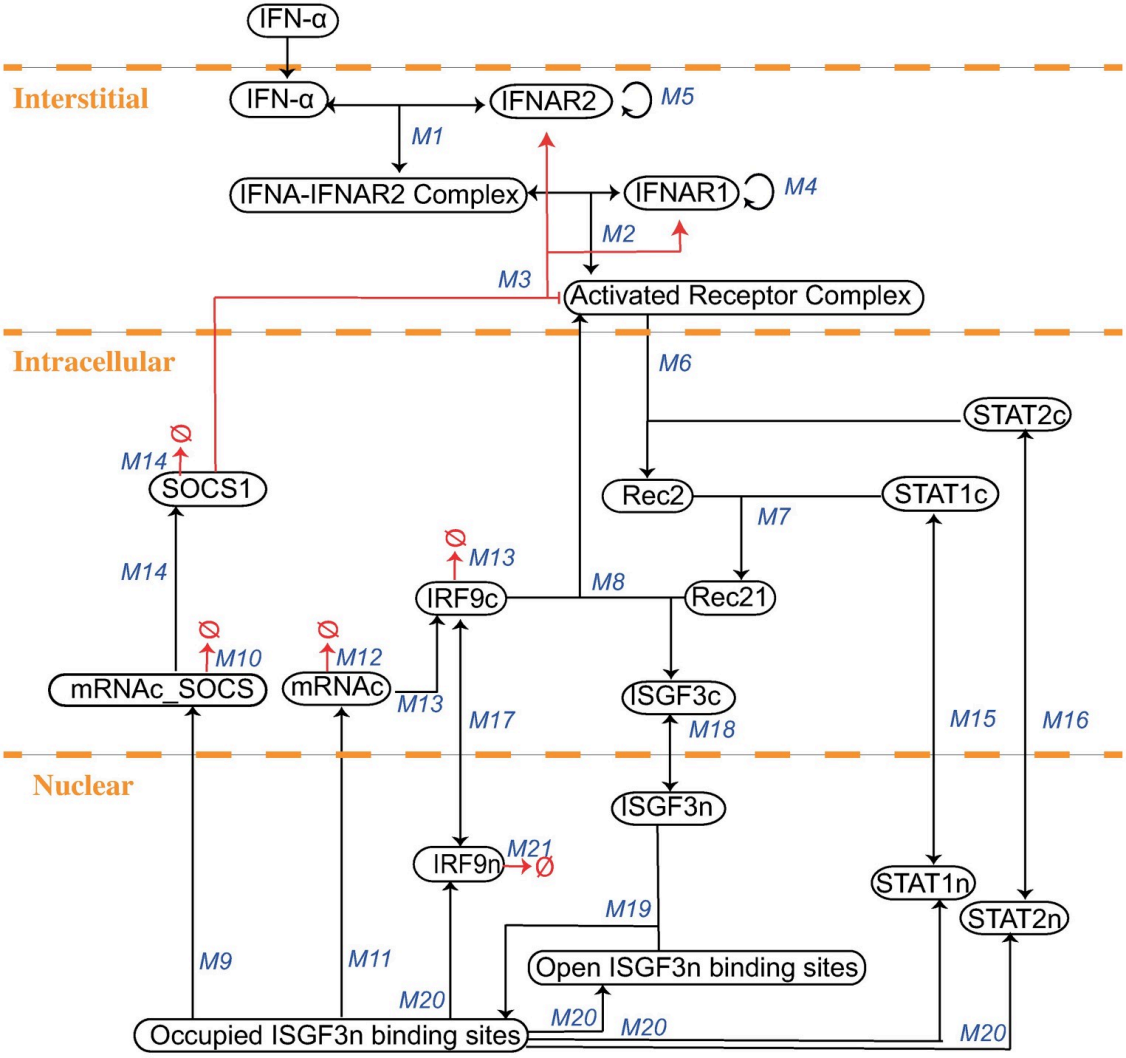
The model schema of the mechanistically based PBPK/PD model details at the liver. The IFN-*α* is cleared via the binding to the respective receptors in the liver. The binding of IFN-*α* to the IFNAR2 and IFNAR1 takes place in the interstitium of the liver (M1-M5), hence activating the JAK/STAT pathway leading to the activation of mRNAc of the response protein IRF9 in the intracellular part of the liver (M6-M14). The reactions in the interstitim constitute the interaction between the PBPK and the intracellular model. Parameterisation is derived from the PBPK model.

The intracellular pathway model was next integrated into the whole-body PBPK model such that, structurally, the intracellular signalling was assumed to happen in the liver compartment only. For this purpose, the intracellular model which was developed using the software COPASI had to be manually transferred to MoBi^®^. To ensure the correctness of the model transfer, we tested for the consistency in two ways. First, the agreement between simulations of the cellular model in both software tools was verified. Second, after the integration simulations with the detailed PBPK/PD model were compared with results of the cellular model for similarity. To this end, the reactions which connect the pharmacokinetic model with the intracellular model were defined in the interstitial compartment of the whole-body model (see Fig 7) and concentrations in interstitial compartment of the PBPK model were set equal to the *in vitro* concentrations. Additionally, the administration of IFN-*α* in the PBPK model was neglected and set to zero to validate the congruence.

### Analysis of the integrated model

The integrated PBPK/PD model of IFN-*α* model allows the simultaneous description of IFN-*α* pharmacokinetics at the whole-body level following intravenous administration and the resulting pharmacodynamic response in the JAK/STAT signalling cascade in an *in vivo* context. The pharmacokinetic behaviour of therapeutic proteins is often influenced by binding to a specific target, i.e. for example an antigen receptor. The model allows quantification of the IFN-*α* receptor induced intracellular PD response and estimates the actual amount of IFN-*α* at the site of action, namely the liver.

**Fig 8:**
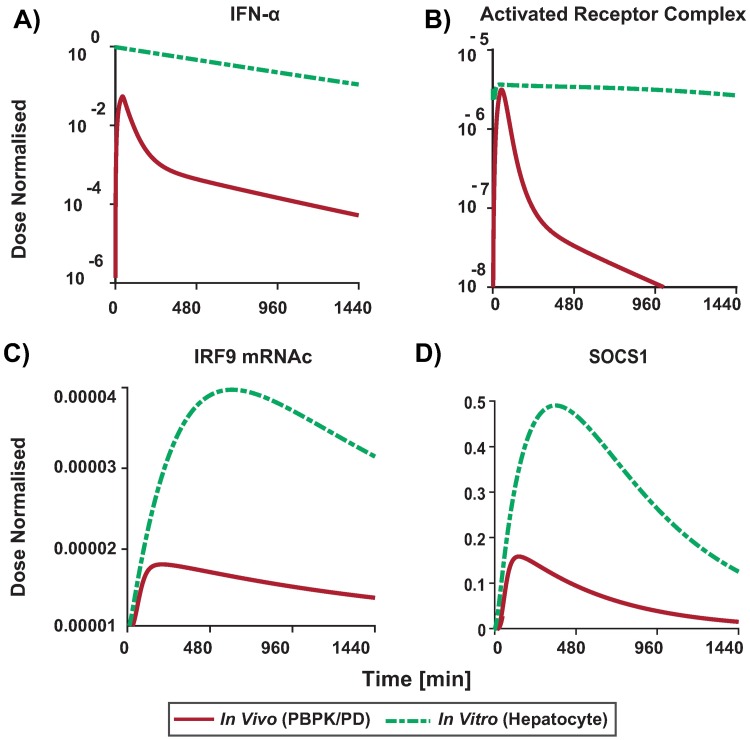
Relative difference in signalling dynamics in the *in vivo* (PBPK/PD) response (to 36U typically administered dose) vs. the *in vitro* (hepatocyte) response (to 500U as typically used in experimental setups). PBPK/PD model (red lines), hepatocyte culture simulation (green lines). Simulation of concentration time profiles in human and in human cell lines of A) the non-linear IFN-*α*, B) activated receptor complex C) IRF9 mRNAc and D) SOCS1 activated downstream in the models.

Next, we simulated the injection of an IFN-*α* dose (36 U) as in [36] with the fully integrated model and followed the behaviour of the intracellular response. In particular, we compared the constant *in vitro* experiment concentration (500 U [20, 45, 49, 50]) to a realistic *in vivo* situation following an i.v. injection of 36 U of IFN-*α* [36] Fig 8. We found that the *in vivo* scenario lead to a C_max_ of 0.7 nmol/l while in the *in vitro* experiments a C_max_ of 13 nmol/l is reached. Obviously, it is to be expected that there are differences in the dynamics of the signalling pattern between *in vivo* and *in vitro* situations and doses (Fig 8B–8D). For example the onset of the *in vitro* response is slightly faster than the *in vivo* response although the C_max_ is reached faster *in vivo*. Interestingly, our simulations suggest the same trend when using the administered dose (C_max_ of 0.7 nmol/l) in the *in vitro* settings (S6 Fig).

**Fig 9:**
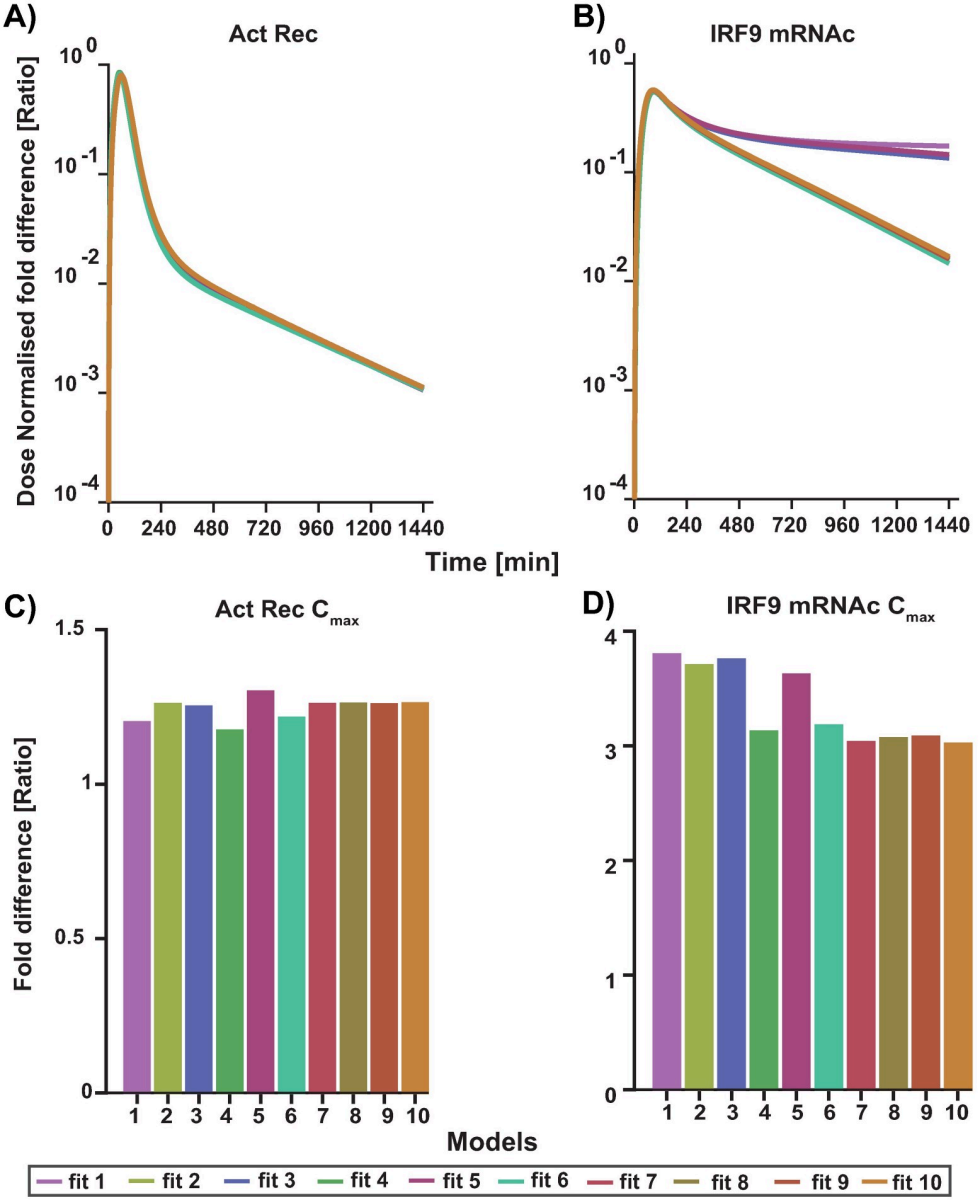
Comparison of cellular signalling responding to typical experimental vs *in vivo* stimulation with IFN-*α*. Intracellular signalling was modelled in response to a constant dose of 500U (as typically used *in vitro*) in comparison to the calculated PBPK profile resulting from a typical dose of 36U. Fold difference is plotted of A) Activated receptor complex and B) IRF9 mRNAc simulation (solid lines) concentration time profile for the top ten models. C) The calculated fold difference C_max_ achieved for activated receptor complex (Act Rec) (bars for each model respectively) and D) fold difference in the C_max_ of IRF9 mRNAc.

The integrated PBPK/PD model allows further mechanistic insights in the dose-response correlation. Our simulation results indicate that the *in vitro* dose are typically too high to be comparable with the *in vivo* situation. However, being close to receptor saturation (Fig 9C) a 18x higher dose (36 U vs 500 U), only results in a 4 fold difference in response, i.e. IRF9 mRNAc (see Fig 9D).

In order to have a comparison of the T_max_ achieved *in vivo* vs *in vitro*, we simulated a constant *in vitro* dose of 0.7 nmol/l (36 U dose) which corresponds to the C_max_ concentration arriving at the liver in our simulation. Because of the lack of drug clearance the *in vitro* behaviour shows a stronger and more sustained response than *in vivo* (S4, S5 and S6 Figs). It is however, interesting to note that despite the numerical differences in dose and cellular response, the shape of the *in vivo* and the *in vitro* PD profile are rather similar (Fig 8C and 8D). In particular, it is worthwhile mentioning that the cellular response is only transient due to negative feedback loops in the JAK/STAT signalling cascade. However, the time scales on which the cellular response reaches its peak is very different in the *in vivo* and the *in vitro* situation, respectively, as reflected by the different T_max_ values.

**Fig 10:**
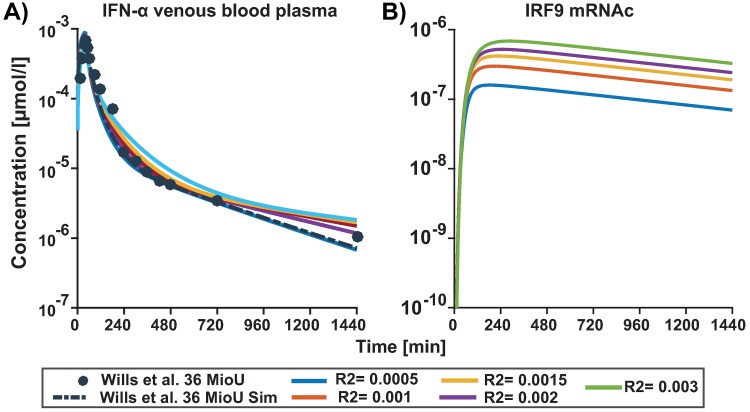
Parameter scan for the initial concentration of IFNA Receptor2. PBPK/PD simulation (lines) of concentration time profile for A) IFN-*α* in venous blood plasma and the experimental blood plasma profile (solid circles) of IFN-*α* in humans. B) IRF9 mRNAc response to different initial concentrations of IFN-*α* Receptor2 that are 0.0005, 0.002, 0.003, 0.0015 and 0.001 *μmol*/*l*. The reference concentration in the initial simulation (dashed black line) for Receptor2 was 0.5 *μmol*/*l*.

Variations in physiological parameters will strongly influence the results. For example receptor ligand binding is one of the essential steps that determines the clearance of IFN-*α* in liver. In our multi-scale PBPK/PD model the influence of this process can be easily assessed by varying the concentrations of IFN-*α* R2 via a parameter scan. Hence, we investigated the impact of receptor abundance and receptor ligand binding on the responses which are not only essential for IFN-*α* clearance by the liver but also will be one of the accountable component of patient variability. We mimicked differences in both receptor abundancy as well as receptor binding (since in the model both parameters will have a correlated effect being part of mass action kinetics). The parameter scan of different concentrations of IFN-*α* receptor 2 (R2) leads to little difference in the plasma ADME of IFN-*α* (Fig 10A) but noticeable difference in response mRNAc concentration profiles (Fig 10B) highlighting the impact of ligand binding to the receptor on the clearance of IFN-*α* at target site (liver) and the notable response difference for the same when considering IRF9 mRNAc as a functional endpoint.

## Discussion

This work presents a multi-scale, mechanistic PBPK/PD model of IFN-*α* drug action. It integrates the whole-body distribution model (data from [36]) with JAK/STAT signalling. In this work we apply a quantitative systems pharmacology approach, to investigate the temporal relationships between IFN-*α* pharmacokinetics and the downstream pharmacodynamic responses in the JAK/STAT pathway. To this end, we developed a multi-scale PBPK/PD model which simultaneously describes two levels of biological organisation: IFN-*α* distribution at the organism scale as well as the resulting cellular response in the JAK/STAT pathway. Taken together this model integration allows the establishment of dose-effect relationships for IFN-*α* at the whole-body level. The resulting model compares *in vitro* drug-responses to therapeutic outcomes in an *in vivo* situation amongst others. The model highlights the fourfold IRF9 mRNAc response difference when comparing typical *in vitro* dose (S3 Fig, see 500U predominantly used in most experiments) to *in vivo* dose of 36U IFN-*α*.

In the past, genome scale network models have been integrated in PBPK models [51, 52]. Also PBPK models have been coupled with simplistic or abstract dynamic cellular network models [53–55]. Semi-mechanistic PK/PD models were built using monkey, mouse and human data [56–59]. Additionally, on the macro-scale, analysis of different routes of injections on IFN-*α* pharmacokinetic is addressed by statistical modelling (a.k.a mixed effect models) [60–63]. Moreover, two compartment PK/PD models on patient populations have been reported in the literature [36, 38, 64–66]. The multi-scale PBPK/PD model presented here is a unique example of linking a detailed cellular signalling model to a whole-body PBPK model using the case of therapeutic protein. Detailed models have the advantage that they allow a direct mechanistic mapping of experimental data and modelling results and therefore the prediction of potential interference with specific points in the system.

*in vitro* assays are routinely used in preclinical drug development as well as in systems biology research in order to understand a cellular response. Doubtlessly, they have been very successful in many respects [67–69]. The results of the PBPK/PD model approach can help to improve the *in vitro* assay design through reverse dosimetry such that the IFN-*α* dose of actual physiological relevance are used in the experiments [70]. As described above, the intracellular signalling is four fold reduced in physiological conditions and displays different dynamics due to *in vivo* drug elimination [71–73].

This is of particular relevance since *in vitro* concentrations are frequently chosen at high and saturating levels like in the case of IFN-*α* where the dose of 500 U is normally used for experiments. This trigger is apparently not reflecting the natural, physiological situation nor the one during clinical usage. We are aware that our intracellular models have been based on such studies and it can be questioned, how close the data gained from cell lines in culture is representing the actual behaviour of cells in a tissue and an *in vivo* environment. However, in [20] there has been at least a comparison between the cell line used and primary hepatocytes showing that the results of the cell lines were very similar both quantitatively and dynamically as compared to primary cells. Again, isolated primary cells are not consistently behaving as they would in their physiological context. So, it is possible that the actual cellular response might be stronger than what we estimate here in this study, e.g. due to paracrine signalling in response to IFN-*α*. However, in the absence of methodology that follows IFN-*α* signalling *in vivo* in a time-dependent manner, this work quantifies the differences between *in vivo* and *in vitro* scenarios. Thus, approaches like this model can contribute to techniques like *in vitro*-*in vivo* extrapolation. The results of the PBPK/PD model approach can help to improve the *in vitro* assay design so that IFN-*α* of actual physiological levels and timings are used. Integration of the pharmacokinetic behaviour with the intracellular signalling is profoundly different from having the separately calculated amount of IFN-*α* in the liver as an administrated dose to the hepatocyte (virtual compartment in the model) and calculating the corresponding response. In the integrated model, both processes occur simultaneously and any time-dependent change in the availability of the drug at the site of action immediately influences the cellular behaviour.

As tissue distribution studies in humans are clinically infeasible, PBPK/PD models as the one presented in this study can be used to quantify tissue exposure and can be compared with animal model experiments. As an add-on, such a model helps to quantify differences between animal studies and human patients. Since target mediated disposition was found to play a major role in the therapeutic efficiency of IFN-*α* treatments, it is furthermore conceivable that PBPK/PD models are individualised through different quantitative phenotypes of antigen presenting individuals.

Another point to be aware of is that the dosages and concentrations of IFN-*α* are given in biological units of its activity. This magnitude is derived from the established activity of a reference standard. These standards however often differ in each study. Thus, it is often difficult to compare doses and serum concentrations quantitatively. Moreover, many studies in the literature do not publish the established activity unit conversions hence leading to constraints for quantitative models.

It should be noted that for the cellular scale a lot of experimental data were available for this work, most of these measurements were from short time scales ranging between 2-6 hours. However, for long-term behaviour much less data is available and therefore the model has limits to quantify the long-term behaviour. However, the important intracellular observations, namely peak amplitude and the speed of activation occur within the short time for which we had a high degree of confidence. For some proteins we could rely on quantitatively calibrated concentration profiles. However, for proteins which are measured without calibrator and only given in relative arbitrary units in the experimental time series, the model is only able to quantify relative changes. This is especially true for the amount of mRNAs simulated. Therefore, we normalised the data and do not work with absolute amounts for these species. In addition, the data used for validation is from experiments that use other subtypes of IFN-*α*. Using different subtype only for the validation ensures that the calibration of the model is not changed as the fitting was done by using only one subtype of IFN-*α* (IFN-*α* 2a). Although, the subtypes may have differences in their specific and kinetic activity, it seems that the qualitative trends in the induced cellular responses are quite similar among the IFN-*α* subtypes.

The PBPK/PD model of IFN-*α* also allowed the derivation of the concentration-effect relationship between IFN-*α* and its response on the functional biomarker IRF9 mRNAc. The model suggests that the saturated response of IRF9 mRNAc is attained at lower IFN-*α* administered dose in comparison to *in vitro* experiments.

A significant observation is that in classical PK/PD models one only infers the dose with simplistic models in combination with plasma concentration levels which are not comparable to the integration of a detailed signalling cascade as undertaken here. This may be in particular true for biologics like IFN-*α* as they are produced by most cells of our body and function in both autocrine and paracrine manner. Hence, such a systemic effect at the whole-body level may only be possible to describe with a multi-scale PBPK/PD modelling approach. Finally, we would like to stress that the described pipeline could be used for any drug eliciting intracellular responses and is by no means restricted to IFN-*α* signalling. Patient specific data can be integrated at the level of protein abundancies, e.g. for the receptors. In the end, for an at least semi-automated set-up of such integrated multi-scale models it would be important to specify a common standard for model exchange between the cellular and pharmacokinetic models.

## Conclusion

In this work we established a multi-scale PBPK/PD model for IFN-*α* combining the pharmacokinetic behaviour of the therapeutic protein after administration to a human and the intracellular signalling elicited by the administered dose. We observed that intracellular responses in physiological conditions is reduced compared with the one reported in cell cultures, albeit only about four fold.

The presented multi-scale PBPK/PD model supports a mechanistic understanding of how a cellular system responds to drug administration. This is of particular relevance, since many new drug therapies are using protein therapies and at the same time envisage personalised treatment regimes for individual patients. This model exemplifies that in the case of therapeutic proteins (biologics), establishing such in-detail models strengthen insights of the dose effect relationships. Due to the ubiquitous presence of such proteins in our bodies, small tweaks at the physiological level can lead to visibly different responses in the cellular pathway. A mechanistic understanding of the complex interplay of a patient’s physiology and mechanistic pharmacodynamics is of primary importance to account for the complex interplay of feedbacks and the biomarkers established to study that. Notably, the presented workflow is generic and therefore not limited to therapeutic proteins [74]. Last but not least, this work highlights the major dynamic dose-effect relationship differences between in physiology vs. cell culture conditions and helps to shed light on the missing aspects and knowledge of the same.

## Supporting information

S1 Text Cellular model description. (PDF)

S2 Text Pharmacokinetic model description. (PDF)

S1 Table Reaction list. (PDF)

S2 Table Description of the kinetic parameter values. (PDF)

S3 Table *k*_*D*_ values found in the literature. (PDF)

S4 Table Values of the literature parameters for the PBPK model of IFN-*α* used in this study. (PDF)

S5 Table Volumes of the liver compartments. (PDF)

S6 Table C_max_ and AUC values. (PDF)

S1 Fig The full scheme of kinetic model of the IFN-*α* signalling pathway. (TIF)

S2 Fig The top ten fits. The top ten fits obtained from the fitting process for 20 datasets is depicted. In the figure the time course profile for A) pStat cytoplasm in response to 500 U B) pSTAT nucleus in response to 500 U C) pSTAT cytoplasm with overexpression of IRF9 protein D) pSTAT nucleus with overexpression of IRF9 protein E) pStat cytoplasm in response to 500 U (second replicate) F) pSTAT nucleus in response to 500U (second replicate) G) IRF9 mRNAc in response to 10U from Bolen et al. [45] H) IRF9 mRNAc in response to 100U from Bolen et al. [45] I) pSTAT total in nucleus in response to 500 U J) pSTAT total in cytoplasm in response to 500U K) pSTAT total in nucleus in response to 1000 U L) pSTAT total in cytoplasm in response to 1000U M) pJak in response to 500U (Activated receptor complex)) N) pSTAT total in nucleus O) IRF9 protein total in nucleus P) mrna socs in response to 500U Q) mrna socs with overexpression of IRF9 protein R) pSTAT total in nucleus in response to 500U S) SOCS protein with overexpression of IRF9 protein T) SOCS protein in response to 500U.
(TIF)

S3 Fig IFN-*α* dose response data on cell lines as found in literature. (TIF)

S4 Fig IRF9 mRNAc T_max_. This table shows the difference in time scale of achieving the maximum concentrations when the IFN-*α* constant dose is simulated as the *in vivo* dose (0.7 nmol/l instead of 13 nmol/l) in the top ten models for PBPK/PD model (*in vivo*) in the liver and in the hepatocyte (*in vitro*) conditions is depicted.
(TIF)

S5 Fig Simulation of the hepatocyte *in vitro* model with the *in vivo* dose (C_max_ 0.7 nmol/l). Relative fold difference of IRF9 mRNAc T_max_ calculated by simulating the typical administered dose of 36U IFN-*α* (C_max_ 0.7 nmol/l) for PBPK/PD and *in vitro* hepatocyte model.
(TIF)

S6 Fig Simulation of IFN-*α* response comparing a constant (green) and a dynamic (red) dose as seen *in vivo* for identical C_max_ of 0.7 nmol/l. Temporal dynamics of A)IFN-*α* B) Activated Receptor Complex C) IRF9 mRNAc D) SOCS.
(TIF)
