# Supplementary material for: Quantitative systems pharmacology of interferon alpha administration: A multi-scale approach
Source: PLoS One. 2019 Feb 13;14(2):e0209587. doi: 10.1371/journal.pone.0209587 (PMC6374012; doi:10.1371/journal.pone.0209587)
Supplement: S1 Text — (PDF) [file pone.0209587.s001.pdf]

S1 Text. Cellular model description. The model is reduced compared to the model by Maiwald et al. [1]. It comprises known key components of the JAK/STAT pathway and consists of 21 species and 20 kinetic reactions. Most of the reactions are described using mass action kinetics. The feedback mechanism as well as transcription has been modelled with more complex reaction kinetics. The proteins located in the cytoplasm or nucleus have the suffix *c* or *n* respectively.

In the following, we briefly describe the structure of the model using the names defined in the model. The corresponding table serves as overview of the reactions (**S1 Table**) and the kinetic parameters (**S2 Table**).

The model simulation time is for 24 hours. The half life of IFN has been observed to be between 4 to 8 hours [2–4]. The degradation of IFN has been included in the cellular model (**R1**) to account for the short life span. The free *IFN* binds to the *IFNAR2* subunit to form a pre *IFNA_R2_Complex* (**R2**), which in turn trimerises with IFNAR1 receptor subunit to form *Activated Receptor Complex* (**R3**). The *Activated Receptor Complex* binds to free cytoplasmic STAT2 to activate the phosphorylated *Rec2* (**R4**) which is followed by binding to free cytoplasmic STAT1 to have the phosphorylated *Rec21* (**R5**).

The phosphorylated dimer *Rec21* associates to *IRF9c* in the cytoplasm to form the *ISGF3c* complex (**R6**). The monomeric subunits of *STAT1c/n* , *STAT2c/n* and *IRF9c/n* are in free exchange between the nucleus and the cytoplasm (**R14-16**). *ISGF3c* complex is actively transported in the nucleus (**R17**) where it binds to the *Open ISGF-3n binding sites* located on the DNA and transcribes the mRNA for SOCS and IRF9; *mRNAc_SOCS* and *mRNAc* respectively (**R18, R8, R10**). The nuclear phosphatase feedback is modelled as a simplistic step where the *Occupied ISGF-3n binding sites* liberates the individual protein of the complex *STAT1n*, *STAT2n*, *IRF9n* and *Open ISGF-3n binding sites* (**R19**).

mRNAc and *mRNAc_SOCS* are degraded (**R9 & R11**). The translation and degradation of the proteins *SOCS1* and *IRF9* are taken into account (**R12 & 13**, **R20**). Finally, the negative feedback loop exerted by *SOCS1* on the receptor has been included (**R4**).

References

1. Maiwald T. Computational analysis of the interferon alpha signalling pathway using a systems biology modelling approach [PhD dissertation]. University of Heidelberg; 2012. Available from: `http://www.ub.uni-heidelberg.de/archiv/13164`.

2. Branca A, Faltynek CR, D'Alessandro SB, Baglioni C. Interaction of interferon with cellular receptors. Internalization and degradation of cell-bound interferon. Journal of Biological Chemistry. 1982;257(22):13291–13296.

3. Arnheiter H, Ohno M, Smith M, Gutte B, Zoon KC. Orientation of a human leukocyte interferon molecule on its cell surface receptor: carboxyl terminus remains accessible to a monoclonal antibody made against a synthetic interferon fragment. Proceedings of the National Academy of Sciences. 1983;80(9):2539–2543.

4. Arnaud P. [The interferons: pharmacology, mechanism of action, tolerance and side effects]. Rev Med Interne. 2002;23 Suppl 4:449s–458s.
