# Supplementary material for: Quantitative systems pharmacology of interferon alpha administration: A multi-scale approach
Source: PLoS One. 2019 Feb 13;14(2):e0209587. doi: 10.1371/journal.pone.0209587 (PMC6374012; doi:10.1371/journal.pone.0209587)
Supplement: S2 Text — (PDF) [file pone.0209587.s002.pdf]

S2 Text. Pharmacokinetic model description.

In this section we discuss the basic assumptions underlying the mathematical description of the pharmacokinetic model. The model parameters and the various literature sources used to describe the model are in tables S1 Table,S2 Table in the supplement.

PBPK reactions. The clinical profile of IFN-α was modelled as zero order input rate and first order elimination rate. All the parameters for the pharmacokinetic modelling are listed in S4 Table. For modelling purposes, the liver is simplified as one big cell which is calculated on the size of the hepato-cellularity of the liver. The liver is divided into 3 compartments: the interstitial, the cytoplasm and the nucleus. The defined volumes of each compartment are seen in Supplementary S5 Table. The IFN receptor dynamics was modelled in the interstitial compartment and consists of detailed receptor ligand interactions along with receptor turnover. The range of biologically valid values of the concentrations of the receptors for parameter estimation was calculated as in 1. Remaining downstream reactions were compartmentalised as they were in the cellular signalling model S1 Table.

1 Calculation of receptor concentration. A mean receptor density of approximately 0.55 molecules/$\mu\ m^2$ has been measured, which corresponds to 500-1000 binding sites per cell [1–4]. The number of hepatocytes per gram of human liver has been estimated as 139 $\times$ 10^6 cells/g liver [5]. It is known that the volume of the liver is 1.6 litres. With this information, total receptor concentration in the liver was calculated.

$$Rec_{exp}(\frac{Receptor}{ml}) = 1000(\frac{Receptor}{cell}) * 139 * 10^6(\frac{gliver}{ml}) \tag{1}$$

$$Rec_{conc}(\frac{mol}{ml}) = \frac{1.48 * 10^{11}(\frac{Receptor}{ml})}{6.02 * 10^{23}(\frac{Receptor}{mol})} \tag{2}$$

$$Rec_{conc}Liver = 2.47 * 10^{-4}(\frac{\mu mol}{l}) * 1.6l \tag{3}$$

$$Rec_{\mu mol}Liver = 3.88 * 10^{-4}\mu mol \tag{4}$$

References

1. Francois-Newton V, Livingstone M, Payelle-Brogard B, Uzé G, Pellegrini S. USP18 establishes the transcriptional and anti-proliferative interferon α/β differential. Biochemical Journal. 2012;446(3):509–516. doi:10.1042/BJ20120541.

2. Wilmes S, Beutel O, Li Z, Francois-Newton V, Richter CP, Janning D, et al. Receptor dimerization dynamics as a regulatory valve for plasticity of type I interferon signaling. Journal of Cell Biology. 2015;209(4):579–593. doi:10.1083/jcb.201412049.

3. Moraga I, Harari D, Schreiber G, Uzé G, Pellegrini S. Receptor density is key to the alpha2/beta interferon differential activities. Molecular and cellular biology. 2009;29(17):4778–87. doi:10.1128/MCB.01808-08.

4. Schreiber G, Piehler J. The molecular basis for functional plasticity in type I interferon signaling. Trends in Immunology. 2015;36(3):139–149. doi:10.1016/j.it.2015.01.002.

5. Sohlenius-Sternbeck AK. Determination of the hepatocellularity number for human, dog, rabbit, rat and mouse livers from protein concentration measurements. Toxicology in Vitro. 2006;20(8):1582 – 1586. doi:http://dx.doi.org/10.1016/j.tiv.2006.06.003.
