# Supplementary material for: Quantitative systems pharmacology of interferon alpha administration: A multi-scale approach
Source: PLoS One. 2019 Feb 13;14(2):e0209587. doi: 10.1371/journal.pone.0209587 (PMC6374012; doi:10.1371/journal.pone.0209587)
Supplement: S1 Table — (PDF) [file pone.0209587.s003.pdf]

S1 Table. Reaction List. The individual reactions for both models: the *in vitro* hepatocyte and the *in vivo* PBPK/PD model. The column with R# and Cell Compartment depict the reactions that are present in the *in vitro* hepatocyte model and where. The columns with M# and multi-scale compartment do the same for the *in vivo* PBPK/PD model. The column reactions describe the modelled pathway along with the column kinetic rate law which gives insight on the kinetics used to model each step.

#		Cell Compartment	Multiscale Compartment	Reaction	Kinetic rate law
R1	-	Cell Intracellular	-	IFN $\rightarrow \varnothing$	$k1 \cdot S$
R2	M1	Cell Intracellular	Liver Interstitial	IFNA + IFNAR2 $\rightleftharpoons$ IFNA-IFNAR 2 Complex	$k1 \cdot S1 \cdot S2 - P/keq$
R3	M2	Cell Intracellular	Liver Interstitial	IFNA_R2_Complex + IFNAR1 $\rightleftharpoons$ Activated Receptor Complex	$k1 \cdot S1 \cdot S2 - P/keq$
R4	M3	Cell Intracellular	Liver Interstitial	Activated Receptor Complex $\rightleftharpoons$ SOCS1 IFNAR1 + IFNAR2	$\frac{V \cdot S \cdot A}{(\mathrm{Kms}+S)(\mathrm{Ka}+A)}$
-	M4	-	Liver Interstitial	$\varnothing \longrightarrow$ IFNAR1	rel_exp_out*AF*f_o*k_t-S*k_t
-	M5	-	Liver Interstitial	$\varnothing \longrightarrow$ IFNAR2	rel_exp_out*AF*f_o*k_t-S*k_t
R5	M6	Cell Intracellular	Liver Intracellular	Activated Receptor Complex + STAT2c $\rightarrow$ Rec2	$k1 \cdot S$
R6	M7	Cell Intracellular	Liver Intracellular	Rec2 + STAT1c $\longrightarrow$ Rec21	$k1 \cdot S$
R7	M8	Cell Intracellular	Liver Intracellular	Rec21 + IRF9c $\longrightarrow$ ISGF$-$3c + Activated Receptor Complex	$k1 \cdot S$
R8	M9	Cell Intracellular	Liver Intracellular	$\varnothing \xrightleftharpoons[\text{binding sites}]{\text{Occupied ISGF}-\text{3n}}$ mRNAc_SOCS	$k1 \cdot M$
R9	M10	Cell Intracellular	Liver Intracellular	mRNAc_SOCS $\longrightarrow \varnothing$	$k1 \cdot S$
R10	M11	Cell Intracellular	Liver Intracellular	$\varnothing \xrightleftharpoons[\text{binding sites}]{\text{Occupied ISGF}-\text{3n}}$ mRNAc	$k1 \cdot M$
R11	M12	Cell Intracellular	Liver Intracellular	mRNAc $\longrightarrow \varnothing$	$k1 \cdot S$
R12	M13	Cell Intracellular	Liver Intracellular	$\varnothing \xrightleftharpoons{\text{mRNAc}}$ IRF9c	$(k_{\mathrm{const}} + k_{\mathrm{act}})M - P \cdot IRF9_{\mathrm{deg}}$
R13	M14	Cell Intracellular	Liver Intracellular	$\varnothing \xrightleftharpoons{mRNAc\_SOCS}$ SOCS 1	$k\_act \cdot M - K\_deg \cdot P$
R14	M15	Exchange	Exchange	STAT1n $\rightleftharpoons$ STAT1c	$k1 \cdot S - P/keq$
R15	M16	Exchange	Exchange	STAT2n $\rightleftharpoons$ STAT2c	$k1 \cdot S - P/keq$
R16	M17	Exchange	Exchange	IRF9n $\rightleftharpoons$ IRF9c	$k1 \cdot S - P/keq$
R17	M18	Exchange	Exchange	ISGF-3c $\rightleftharpoons$ ISGF-3n	$k1 \cdot S$
R18	M19	Cell Nucleus	Liver Nucleus	Open ISGF-3n binding sites + ISGF-3n $\rightleftharpoons$ Occupied ISGF-3n binding sites	$k1 \cdot S1 \cdot S2 - P/keq$
R19	M20	Cell Nucleus	Liver Nucleus	Occupied ISGF-3n binding sites $\rightarrow$ STAT 1n + STAT 2n + IRF 9n + Open ISGF-3n binding sites	$k1 \cdot S$
R20	M21	Cell Nucleus	Liver Nucleus	IRF9n $\longrightarrow \varnothing$	$k1 \cdot S$
