# Supplementary material for: Quantitative systems pharmacology of interferon alpha administration: A multi-scale approach
Source: PLoS One. 2019 Feb 13;14(2):e0209587. doi: 10.1371/journal.pone.0209587 (PMC6374012; doi:10.1371/journal.pone.0209587)
Supplement: S2 Table — (PDF) [file pone.0209587.s004.pdf]

S2 Table. Description of the kinetic parameter values. The below table is an exemplary representation of all the kinetic parameters obtained for the first model in the model ensemble. The receptor values were preserved from the PBPK model (S2 Table) and the value of IFN efflux was also fitted and kept constant (S2 Table). Most of the values are varied +- 50% or 75% as those used in Maiwald et al [1] or the literature values found for some parameters as stated in S3 Table. The rest of the nine models are provided as SBML files. The kinetic parameters whose values were fixed w.r.t. the PBPK model in the hepatocyte model are shown in the short second table.

**Nr.**	**Parameter**	**fit 1**	**Units**
R4 & M3	Kms	6.13131	nmol/l
	V	2.61285	nmol/(l*s)
	Ka	200	nmol/l
R5 & M6	k1	0.0035	l/(nmol*s)
R6 & M7	k1	0.000393	l/(nmol*s)
R7 & M8	k1	10	l/(nmol*s)
R8 & M9	k1	0.00691842	1/s
R9 & M10	k	13.04024e-05	1/s
R10 & M11	k	1.34948e-07	nmol/(l*s)
	Ka	0.0044274	nmol/l
R11 & M12	k1	1.17183e-05	1/s
R12 & M13	irf9deg	1.039e-05	1/s
	k_const	4.50e-06	nmol/(l*s)
	k_act	8.95	1/s
R13 & M14	k_act	3.0968	1/s
	k_deg	0.0888526	1/s
R14 & M15	k1	0.0001	l/s
	keq	0.00697504	-
R15 & M16	k1	2.20533e-07	l/s
	keq	0.00197389	-
R16 & M17	k1	9.92815e-07	l/s
	keq	0.0297375	-
R17 & M18	k1	0.00774342	l/s
R18 & M19	k1	0.123072	l/(nmol*s)
	keq	2.91162e-05	l/nmol
R19 & M20	k1	0.10365	1/s
R20 & M21	k1	1.039e-05	1/s

**Nr.**	**Parameter**	**Value**	**Unit**
R1	k1	2.57504e-05	1/s
R2	k1	0.1	l/(nmol*s)
	keq	1.5	l/nmol
R3	k1	0.00022	l/(nmol*s)
	keq	0.00077	l/nmol

References

1. Maiwald T, Schneider A, Busch H, Sahle S, Gretz N, Weiss TS, et al. Combining theoretical analysis and experimental data generation reveals IRF9 as a crucial factor for accelerating $IFN-\alpha$-induced early antiviral signalling. FEBS Journal. 2010;277(22):4741–4754. doi:10.1111/j.1742-4658.2010.07880.x.
