# Supplementary material for: Quantitative systems pharmacology of interferon alpha administration: A multi-scale approach
Source: PLoS One. 2019 Feb 13;14(2):e0209587. doi: 10.1371/journal.pone.0209587 (PMC6374012; doi:10.1371/journal.pone.0209587)
Supplement: S3 Table — (PDF) [file pone.0209587.s005.pdf]

S3 Table. k_D **values found in the literature.**

**Molecule**	**Param**	k_D **(nM)**	**Ref.**
ISGF3c	k_D	$[10nM(STAT2), 5\mu M(STAT1)]$	[1]
IFN-IFNR2	k_D	$[2.2nM]*$	[2] [3]
IFN-IFNR1	k_D	$[2000nM]$	[2] [3]
STAT1-STAT2	k_D	$[< 10nM, 5.5mM(STAT2mutant)]$	[4]
SOCS **	k_i	$[1.5 \pm 0.7\mu M(ForJAK2), 1.2 \pm 0.3\mu M(ForATP)]$	[5]

*IFNAR2 binds IFN-α2 with an equilibrium dissociation constant of around 5 nM and a complex lifetime of around 100 s, whereas the affinity toward IFNAR1 is three orders of magnitude lower, and the complex dissociates about 100-fold faster.

** Non-competitive Inhibition

$k_{eq} = 1/k_D$

References

1. Rengachari S, Groiss S, Devos J, Caron E, Grandvaux N, Panne D. Structural Basis Of STAT2 Recognition By IRF9 Reveals Molecular Insights Into ISGF3 Function. bioRxiv. 2017;doi:10.1101/131714.

2. Levin D, Harari D, Schreiber G. Stochastic Receptor Expression Determines Cell Fate upon Interferon Treatment. Molecular and Cellular Biology. 2011;31(16):3252–3266. doi:10.1128/MCB.05251-11.

3. You C, Marquez-Lago TT, Richter CP, Wilmes S, Moraga I, Garcia KC, et al. Receptor dimer stabilization by hierarchical plasma membrane microcompartments regulates cytokine signaling. Science Advances. 2016;2(12):1–13. doi:10.1126/sciadv.1600452.

4. Ho J, Pelzel C, Begitt A, Mee M, Elsheikha HM, Scott DJ, et al. STAT2 Is a Pervasive Cytokine Regulator due to Its Inhibition of STAT1 in Multiple Signaling Pathways. PLoS Biology. 2016;14(10). doi:10.1371/journal.pbio.2000117.

5. Babon JJ, Kershaw NJ, Murphy JM, Varghese LN, Laktyushin A, Young SN, et al. Suppression of cytokine signaling by SOCS3: characterization of the mode of inhibition and the basis of its specificity. Immunity. 2012;36(2):239–250.
