# Supplementary material for: Quantitative systems pharmacology of interferon alpha administration: A multi-scale approach
Source: PLoS One. 2019 Feb 13;14(2):e0209587. doi: 10.1371/journal.pone.0209587 (PMC6374012; doi:10.1371/journal.pone.0209587)
Supplement: S4 Table — (PDF) [file pone.0209587.s006.pdf]

S4 Table. Values of the literature parameters for the PBPK model of IFN-α used in this study.

Parameter	Fitted Values	Reference Value	Unit	Reference
Molecular Weight	19500(fixed)	19500	g/mol	[1]
CLplasma (Kidney)	0.003	0.0027	l/min/kg organ	[2–5]
IFNAR1 Concentration	0.26	-	$\mu mol/l$	[6–9]
IFNAR2 Concentration	0.5	-	$\mu mol/l$	[6–9]
K(IFNAR1)	0.1	-	l/nmol*s	-
K(IFNAR2)	0.00022	-	l/nmol*s	-
K_{eq}IFNAR1	0.00077	0.0001-0.01	l/nmol	[10, 11]
K_{eq}IFNAR2	1.5	0.002-0.1	l/nmol	[12, 13]
$K_{endocytosis}$	8.93226	-	1/min	-
K_t_IFNAR1	0.02298	-	1/min	-
K_t_IFNAR2	0.074999	-	1/min	-

References

1. Wishart DS, Knox C, Guo AC, Shrivastava S, Hassanali M, Stothard P, et al. DrugBank: a comprehensive resource for in silico drug discovery and exploration. Nucleic acids research. 2006;34(Database issue):D668–72. doi:10.1093/nar/gkj067.

2. Wills RJ, Dennis S, Spiegel HE, Gibson DM, Nadler PI. Interferon kinetics and adverse reactions after intravenous, intramuscular, and subcutaneous injection. Clinical Pharmacology & Therapeutics. 1984;35:722–727.

3. Bocci V, Pacini A, Muscettola M, Paulesu L, Pessina GP, Santiano M, et al. Renal filtration, absorption and catabolism of human alpha interferon. Journal of interferon research. 1981;1:347–352. doi:10.1089/jir.1981.1.347.

4. Tamar Bino AG Zacharai Madar, Rosenberg H. The Kidney is the Main Site of Interferon Degradation. Journal of Interferon Research. 1982;2:301–308. doi:10.1089/jir.1982.2.301.

5. Bino T GA Edery H, H R. Involvement of the kidney in catabolism of human leukocyte interferon. The Journal of general virology. Mar 1982;59(Pt 1):39–45. doi:10.1099/0022-1317-59-1-39.

6. François-Newton V, de Freitas Almeida GM, Payelle-Brogard B, Monneron D, Pichard-Garcia L, Piehler J, et al. USP18-based negative feedback control is induced by type I and type III interferons and specifically inactivates interferon α response. PLoS ONE. 2011;6(7). doi:10.1371/journal.pone.0022200.

7. Wilmes S, Beutel O, Li Z, Francois-Newton V, Richter CP, Janning D, et al. Receptor dimerization dynamics as a regulatory valve for plasticity of type I interferon signaling. Journal of Cell Biology. 2015;209(4):579–593. doi:10.1083/jcb.201412049.

8. Moraga I, Harari D, Schreiber G, Uzé G, Pellegrini S. Receptor density is key to the alpha2/beta interferon differential activities. Molecular and cellular biology. 2009;29(17):4778–87. doi:10.1128/MCB.01808-08.

9. Schreiber G, Piehler J. The molecular basis for functional plasticity in type I interferon signaling. Trends in Immunology. 2015;36(3):139–149. doi:10.1016/j.it.2015.01.002.

10. Gavutis M, Jaks E, Lamken P, Piehler J. Determination of the Two-Dimensional Interaction Rate Constants of a Cytokine Receptor Complex. Biophysical Journal. 2006;90(9):3345 – 3355. doi:http://dx.doi.org/10.1529/biophysj.105.072546.

11. Lavoie TB, Kalie E, Crisafulli-Cabatu S, Abramovich R, DiGioia G, Moolchan K, et al. Binding and activity of all human alpha interferon subtypes. Cytokine. 2011;56(2):282–289. doi:10.1016/j.cyto.2011.07.019.

12. Cutrone EC, Langer JA. Contributions of cloned type I interferon receptor subunits to differential ligand binding. FEBS Letters. 1997;404(2-3):197–202. doi:10.1016/S0014-5793(97)00129-4.

13. Rengachari S, Groiss S, Devos J, Caron E, Grandvaux N, Panne D. Structural Basis Of STAT2 Recognition By IRF9 Reveals Molecular Insights Into ISGF3 Function. bioRxiv. 2017;doi:10.1101/131714.
