# Supplementary material for: Quantitative systems pharmacology of interferon alpha administration: A multi-scale approach
Source: PLoS One. 2019 Feb 13;14(2):e0209587. doi: 10.1371/journal.pone.0209587 (PMC6374012; doi:10.1371/journal.pone.0209587)
Supplement: S5 Table — (PDF) [file pone.0209587.s007.pdf]

S5 Table. Volumes of the liver compartments.

Volume in Litres	
**Compartment**	l
Interstitial	0.39
Cytoplasm	1.59
Nucleus	0.35
