# Supplementary material for: Quantitative systems pharmacology of interferon alpha administration: A multi-scale approach
Source: PLoS One. 2019 Feb 13;14(2):e0209587. doi: 10.1371/journal.pone.0209587 (PMC6374012; doi:10.1371/journal.pone.0209587)
Supplement: S6 Table — (PDF) [file pone.0209587.s008.pdf]

S6 Table. $\mathbf{C_{max}}$ and AUC values.

Publication	Dose (U)	Cmax (pg/ml)		AUC $^{0 \rightarrow \infty}$ $(pg/ml \times hr)$	
		**Observed**	**Predicted**	**Observed**	**Predicted**
Wills et al. (1984) [1]	36×10^6	13900 ±3570	15759	17600 ±4430	16236
Shah et al. (1984) [2]	30×10^6	not defined	11708	not defined	10010
	60×10^6		22581		19825
Radwanski et al. (1987) [3]	8.6×10^6 †	855	3864	945	3315

† where the body surface area is assumed to be 1.72 m^2

References

1. Wills RJ, Dennis S, Spiegel HE, Gibson DM, Nadler PI. Interferon kinetics and adverse reactions after intravenous, intramuscular, and subcutaneous injection. Clinical Pharmacology & Therapeutics. 1984;35:722–727.

2. Shah I, Band J, Samon HM, Young J, Robinson R, Bailey B, et al. Pharmacokinetics and tolerance of intravenous and intramuscular recombinant alpha 2 interferon in patients with malignancies. American journal of hematology. 1984;17:363–71.

3. Radwanski E, Perentesis G, Jacobs S, Oden E, Affrime M, Symchowicz S, et al. Pharmacokinetics of Interferon Î±-2b in Healthy Volunteers. The Journal of Clinical Pharmacology. 1987;27(5):432–435. doi:10.1002/j.1552-4604.1987.tb03044.x.
