# Supplementary figures and images for: Quantitative systems pharmacology of interferon alpha administration: A multi-scale approach
Source: PLoS One. 2019 Feb 13;14(2):e0209587. doi: 10.1371/journal.pone.0209587 (PMC6374012; doi:10.1371/journal.pone.0209587)

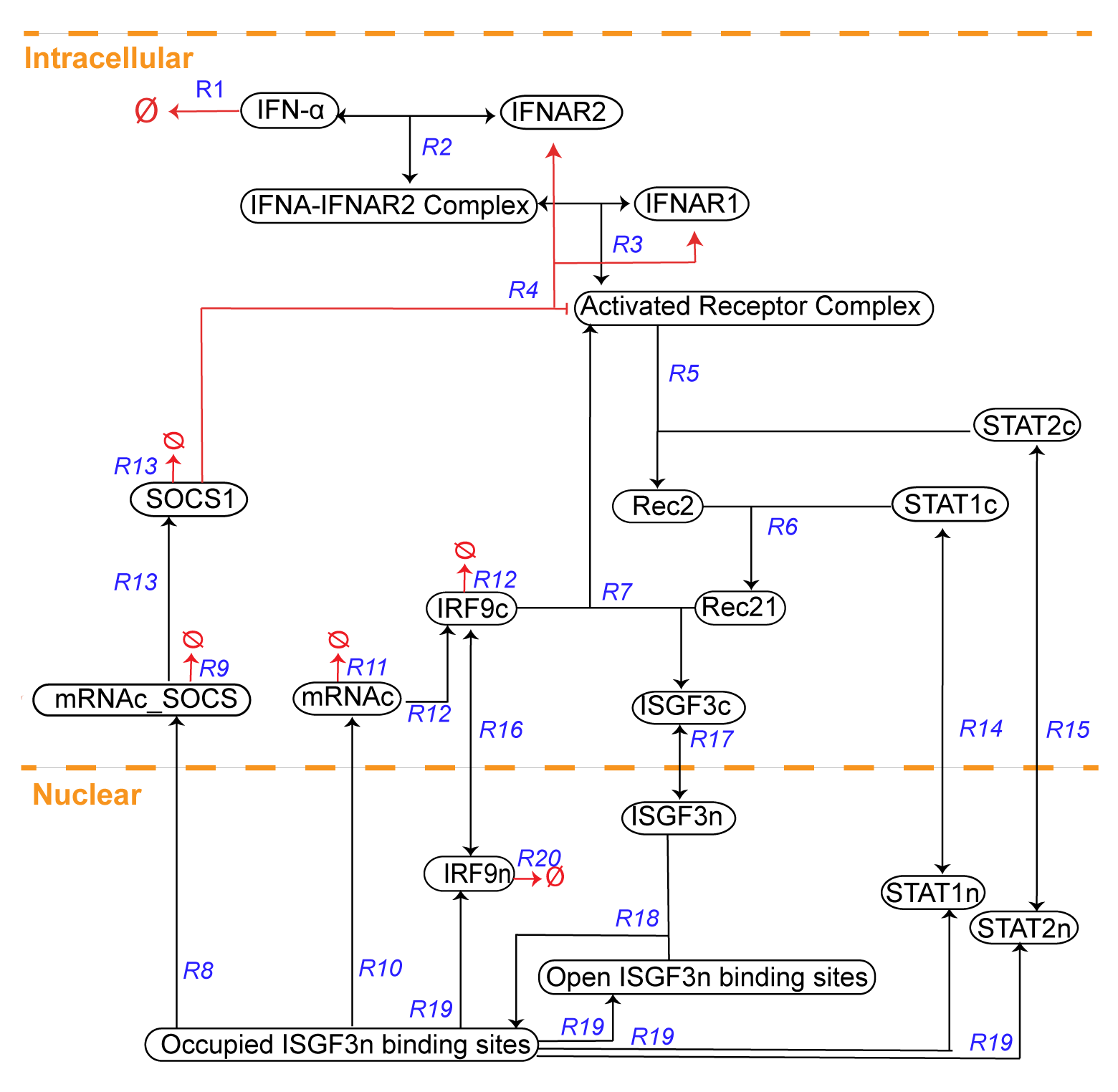

Supplement: S1 Fig — (TIF) [file pone.0209587.s009.tif]

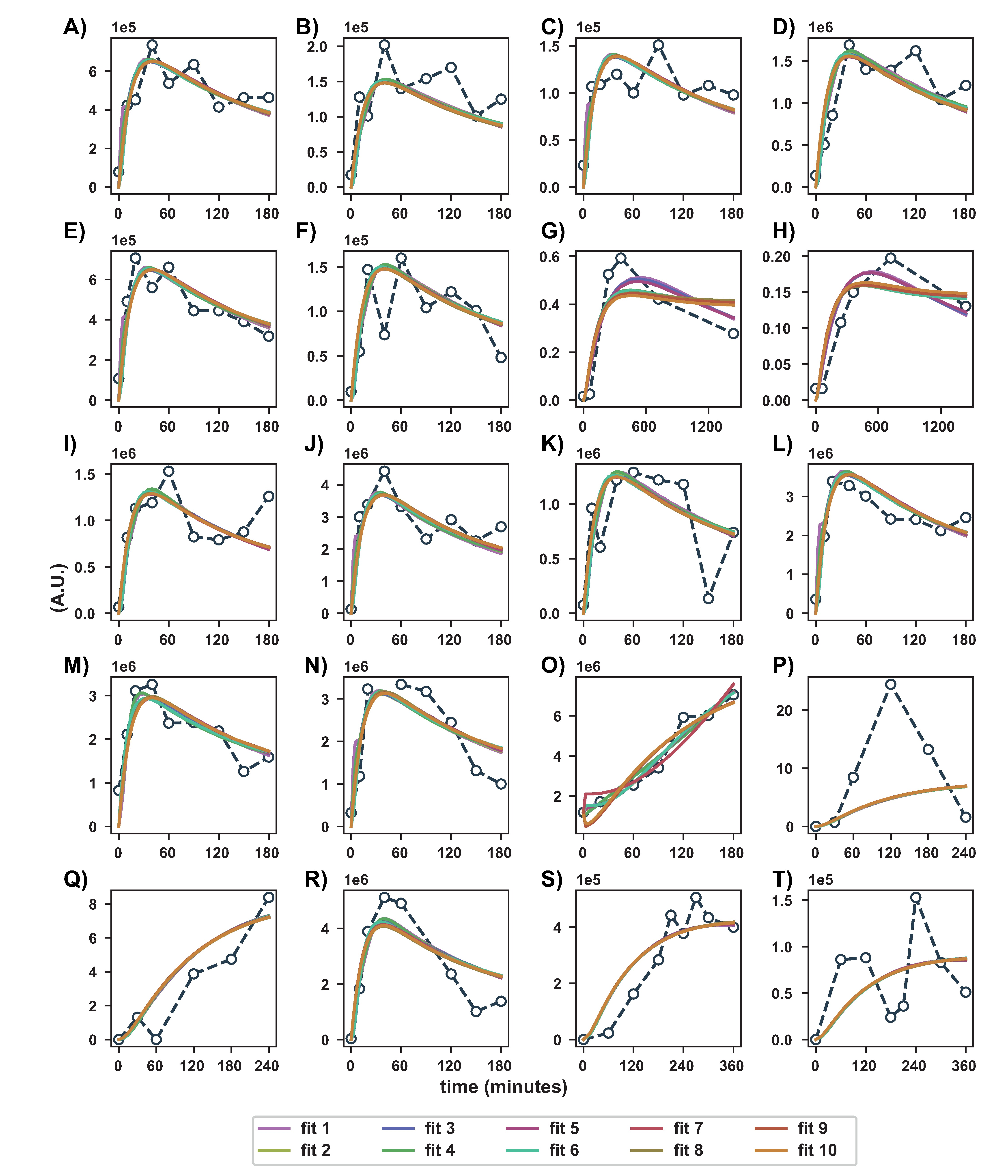

Supplement: S2 Fig — The top ten fits obtained from the fitting process for 20 datasets is depicted. In the figure the time course profile for A) pStat cytoplasm in response to 500 U B) pSTAT nucleus in response to 500 U C) pSTAT cytoplasm with overexpression of IRF9 protein D) pSTAT nucleus with overexpression of IRF9 protein E) pStat cytoplasm in response to 500 U (second replicate) F) pSTAT nucleus in response to 500U (second replicate) G) IRF9 mRNAc in response to 10U from Bolen et al. [45] H) IRF9 mRNAc in response to 100U from Bolen et al. [45] I) pSTAT total in nucleus in response to 500 U J) pSTAT total in cytoplasm in response to 500U K) pSTAT total in nucleus in response to 1000 U L) pSTAT total in cytoplasm in response to 1000U M) pJak in response to 500U (Activated receptor complex)) N) pSTAT total in nucleus O) IRF9 protein total in nucleus P) mrna socs in response to 500U Q) mrna socs with overexpression of IRF9 protein R) pSTAT total in nucleus in response to 500U S) SOCS protein with overexpression of IRF9 protein T) SOCS protein in response to 500U. (TIF) [file pone.0209587.s010.tif]

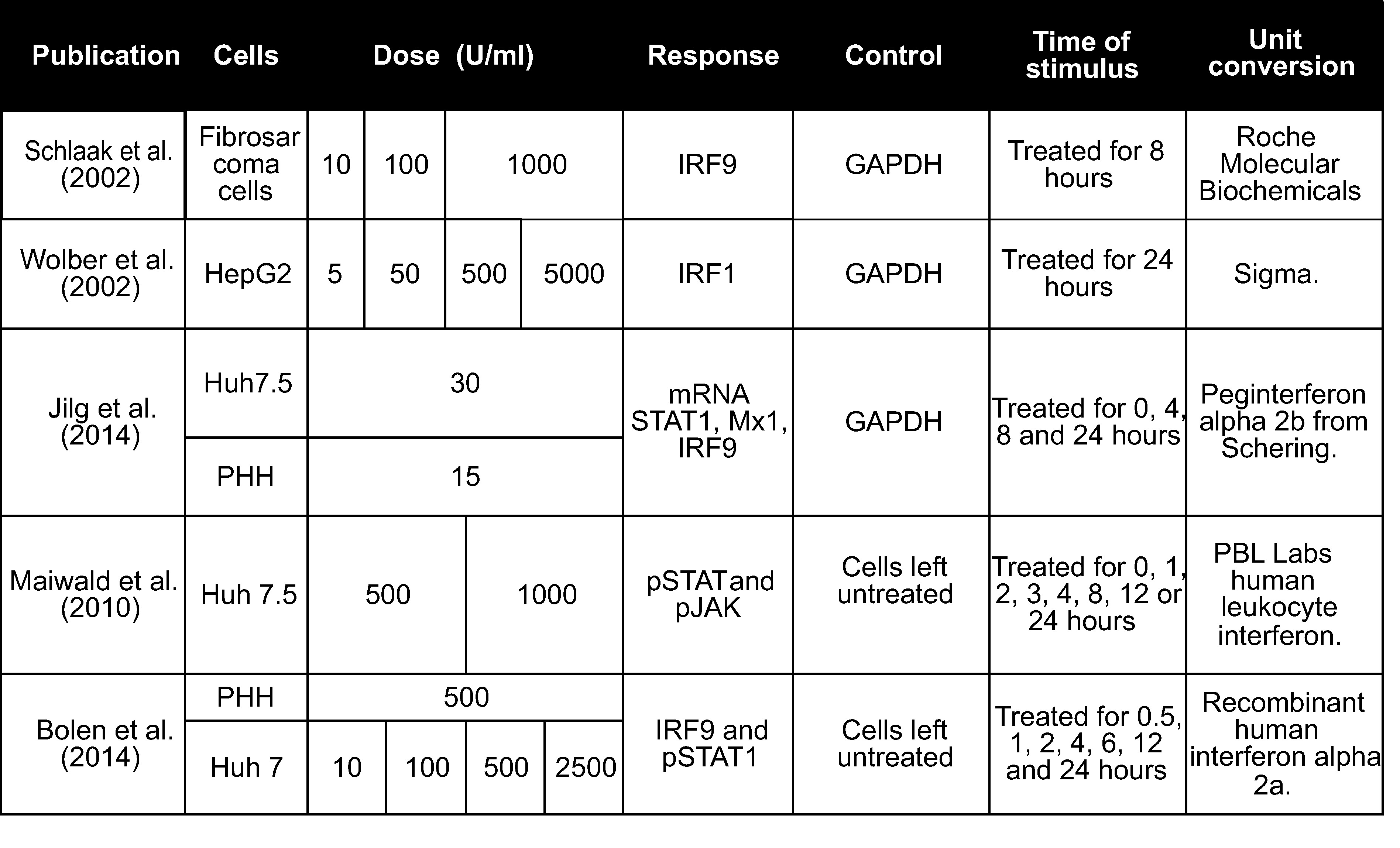

Supplement: S3 Fig — (TIF) [file pone.0209587.s011.tif]

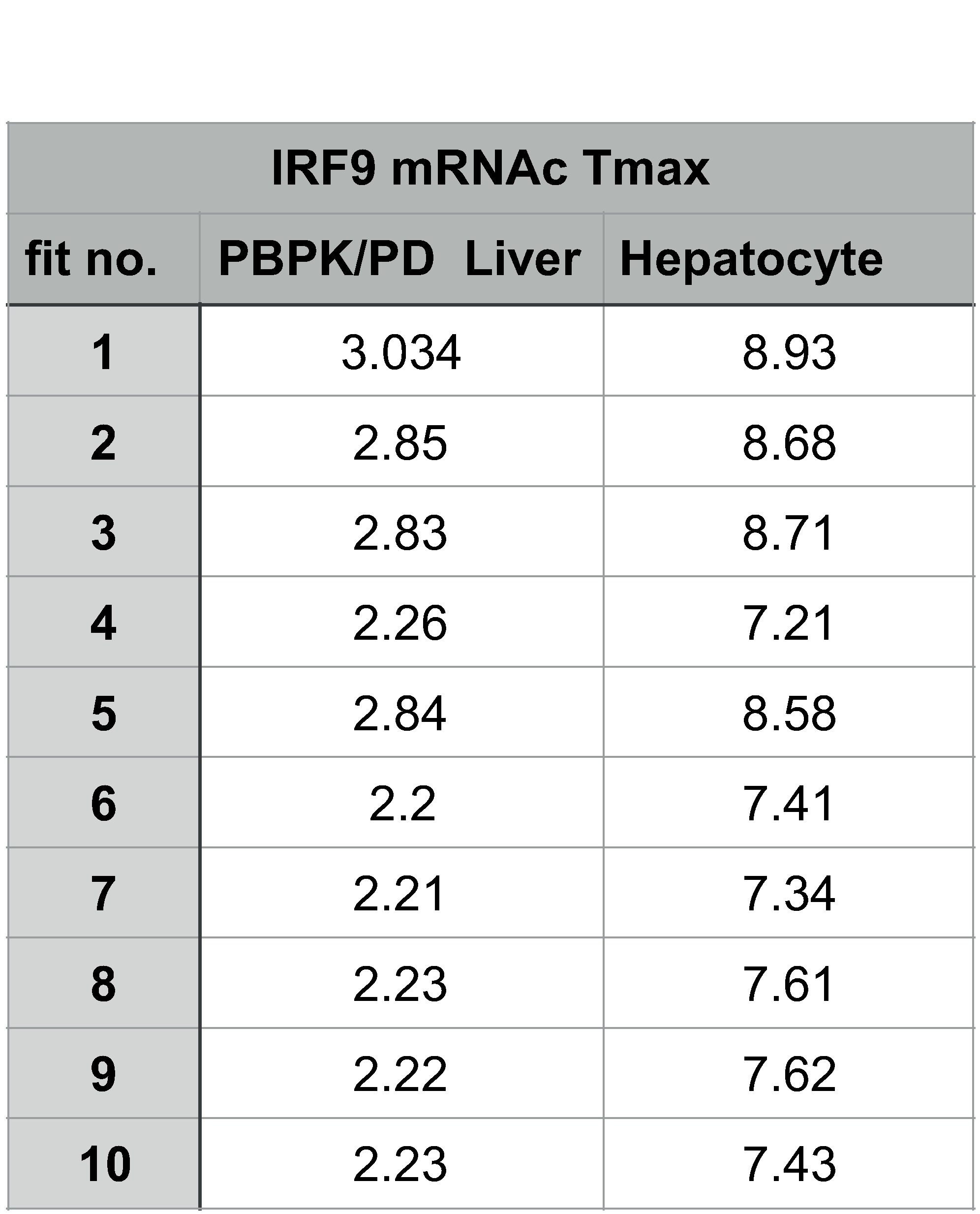

Supplement: S4 Fig — This table shows the difference in time scale of achieving the maximum concentrations when the IFN-α constant dose is simulated as the in vivo dose (0.7 nmol/l instead of 13 nmol/l) in the top ten models for PBPK/PD model (in vivo) in the liver and in the hepatocyte (in vitro) conditions is depicted. (TIF) [file pone.0209587.s012.tif]

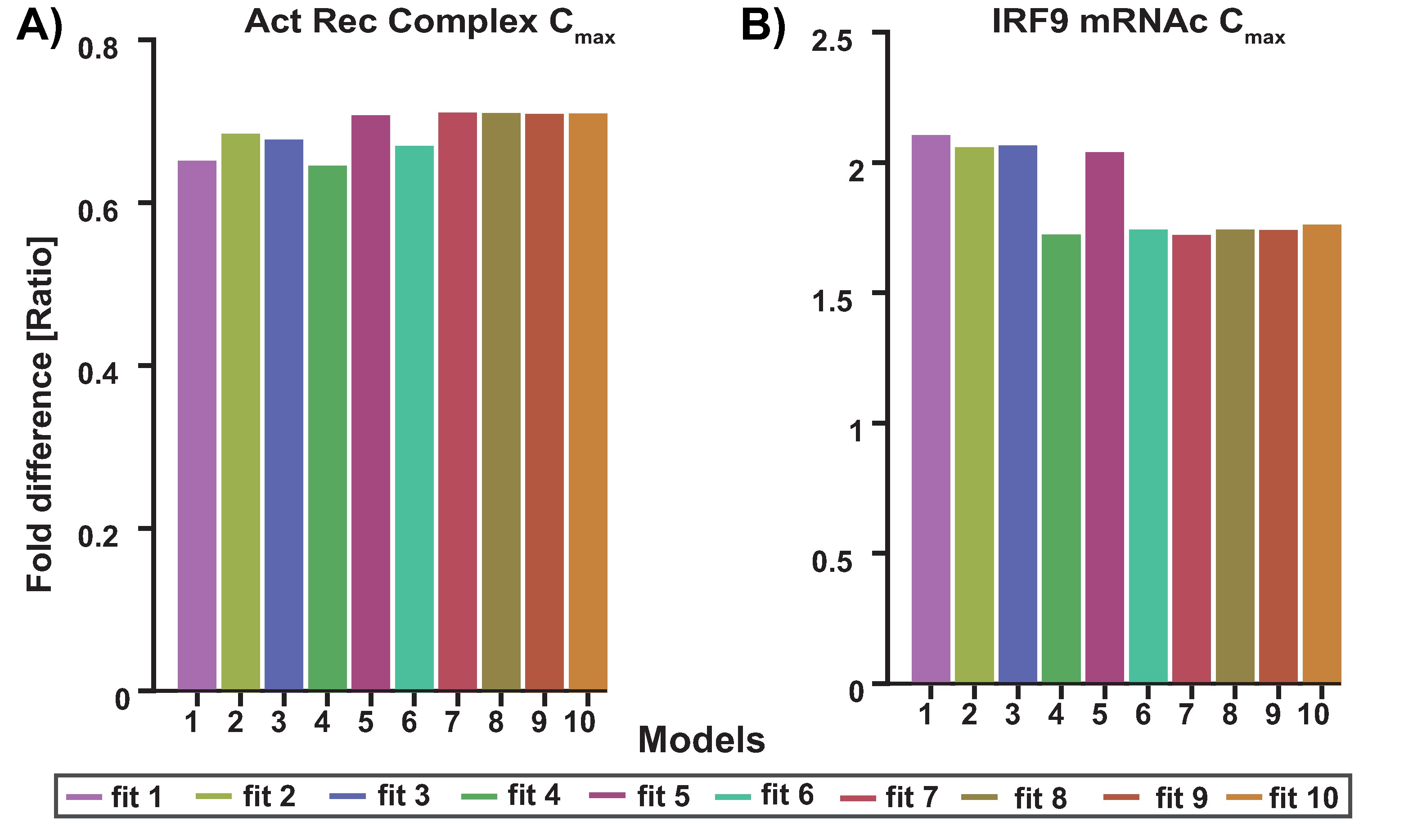

Supplement: S5 Fig — Relative fold difference of IRF9 mRNAc Tmax calculated by simulating the typical administered dose of 36U IFN-α (Cmax 0.7 nmol/l) for PBPK/PD and in vitro hepatocyte model. (TIF) [file pone.0209587.s013.tif]

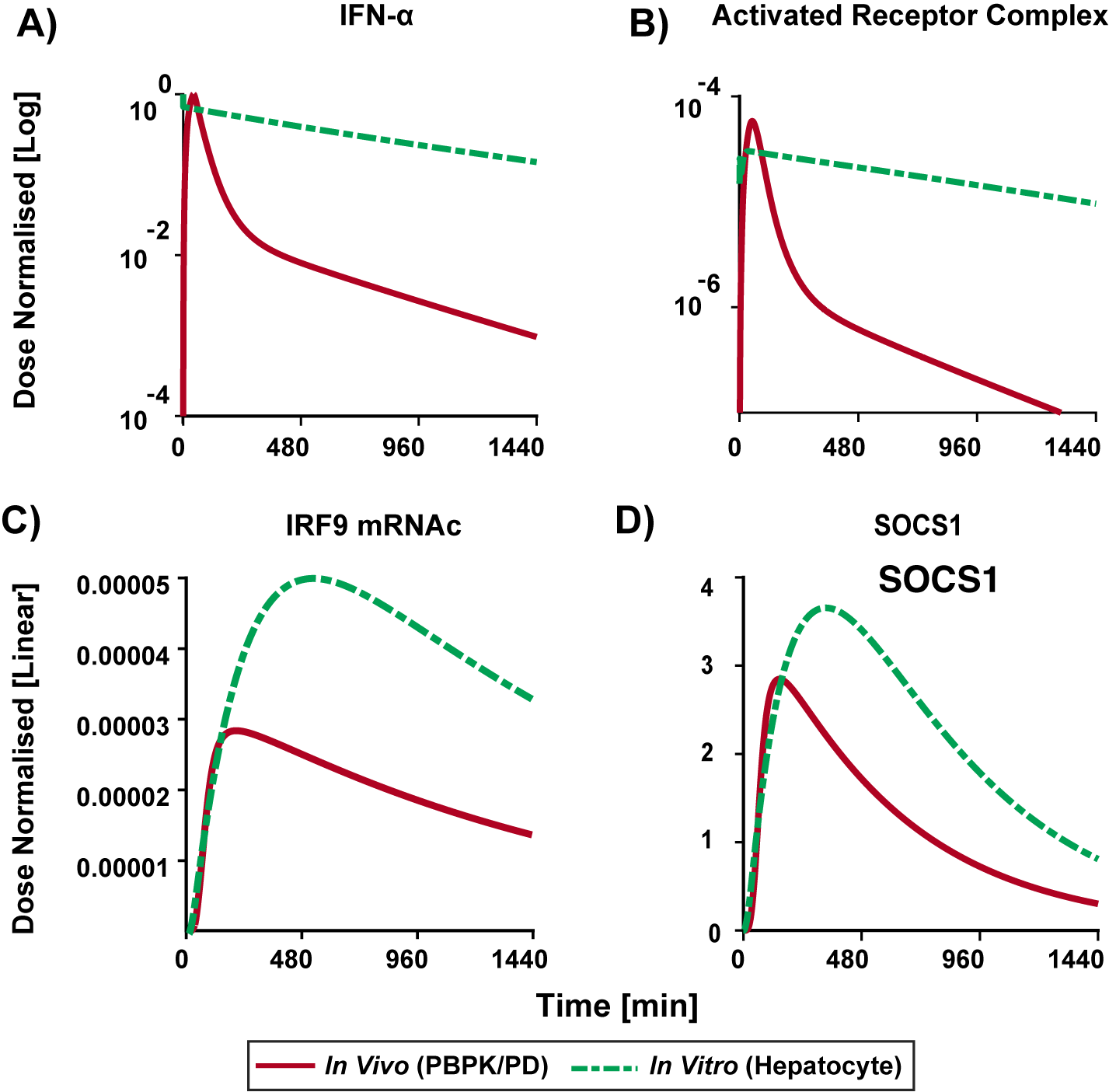

Supplement: S6 Fig — Temporal dynamics of A)IFN-α B) Activated Receptor Complex C) IRF9 mRNAc D) SOCS. (TIF) [file pone.0209587.s014.tif]
